# Sustainability evaluation of sports tourism using a linguistic neutrosophic multi-criteria decision-making method

**DOI:** 10.1371/journal.pone.0300341

**Published:** 2024-03-18

**Authors:** Zhenyin Chen, Suizhi Luo, Feng Zheng

**Affiliations:** 1 Department of Physical Education, Central South University, Changsha, China; 2 College of Tourism, Hunan Normal University, Changsha, China; Vietnam National University, VIET NAM

## Abstract

Sports tourism represents a novel industrial manifestation of the profound integration between the tourism and sports sectors. The objective of this research is to examine an innovative multi-criteria decision-making (MCDM) method for the sustainability evaluation of sports tourism. The largest innovations are the expression and treatment of ambiguous data and interdependent evaluation criteria in the sports tourism sustainability evaluation process. On the one hand, intricate assessment data is represented using linguistic neutrosophic numbers (LNNs), which employ three linguistic variables to convey uncertainty and imprecision. On the other hand, to effectively capture the interrelationships among inputs, two novel aggregation operators are proposed. They are devised based on the Einstein operations and Heronian mean operators of LNNs. Subsequently, a linguistic neutrosophic evaluation method utilizing the aforementioned operators is presented. Comparative and sensitivity analyses conclude that great interdependence exists among five different dimensions of sustainability evaluation in sports tourism, and the proposed method can reflect the interrelations among inputs without redundant calculations.

## Introduction

Nowadays, adhering to the concept of green and healthy sustainable development is the only way for social development. Sustainable development is defined as the development that meets the needs of contemporary people without harming future generations to meet their needs [1]. The proposition of sustainable development is mainly to coordinate the conflict between economic growth and environmental protection. Many scholars introduced the idea of sustainable development into tourism industry [2, 3].

Sports tourism is a series of economic activities that take sports as the core, take on-site watching, participating in experience and visiting as the main form, and provide related products and services to the public for the purpose of satisfying health, entertainment and tourism leisure [4]. Vigorously developing sports tourism is an important measure to promote the deep integration of national fitness and national health, expand tourism consumption space and enrich tourism product system. With the improvement of people’s living standards and the rise of leisure tourism, experience tourism has become a popular way of tourism among tourists. However, it is followed by the destruction of the environment and the waste of resources. Furthermore, with the continuous development of sports tourism, the dependence on more respects (such as the sociality, environment and related technology) is gradually increasing [5]. Nevertheless, there is still a lack of comprehensive and specific evaluation system and methods for the sustainability assessment of sports tourism. Therefore, it is of great importance and urgency to develop new decision-making models specifically for the characteristics of sports tourism sustainability evaluation.

Even though some techniques have been utilized to investigate tourism evaluation with sustainable development, there are still some limitations and challenges, as follows:

1. Three consensual dimensions of sustainability (i.e., social, economic and environmental sustainability) are widely discussed in existing literature. However, some other influencing factors are also very important and need to be considered during the tourism sustainability evaluation process. For instance, sustainability-related institutions and technologies also have great influence on the sustainable development of sports tourism.
2. The evaluation dimensions of sustainability are not completely independent, but usually associated with each other in reality. For example, economic development may promote social equity and welfare; the improvement of tourism service level may stimulate demand and economic growth of tourist destinations; and economic growth may have positive or negative impacts on the environment. Nevertheless, limited researches have taken the correlations among these dimensions into account.

To fill these research gaps, this study intends to establish a new linguistic neutrosophic decision-making (MCDM) model with multiple evaluation criteria. The major motivations are:

1. Because of the ambiguity of human thought and the complicacy of sustainability evaluation, linguistic phrases, like “very high” and “very low”, are frequently utilized to express people’s opinions or attitudes in reality [6]. Linguistic neutrosophic numbers (LNNs) are suggested to describe fuzzy evaluation information in this study. Compared with other linguistic extensions, the main benefit of LNNs is that they can fully reflect decision makers’ truth, hesitancy and false membership degrees with three linguistic phrases. For example, if experts evaluated a *“high”* environmental sustainability of an ice and snow sports attractions based on their knowledge, but they have a *“medium”* hesitancy about the judgment because of dissimilar opinions among different decision makers, and simultaneously there is a *“low”* probability of making a false judgment, then they can expressed this result with a linguistic neutrosophic number *(high*, *medium*, *low)*. This is a common scenario in the real-world evaluation process. In this case, different attitudes or opinions of experts can be simultaneously collected and then the overall evaluation results can be expressed with LNNs.
2. The determination of evaluation criteria is the premise of effective assessment, which directly affects the decision-making results. As mentioned earlier, the sustainable development of sports tourism is not only related to sociality, economy and environment. More influence factors or evaluation dimensions, such as the institution and technology, need to be identified and a more comprehensive assessment index system should be established.
3. Owing to multiple dimensions embodied in the sustainability evaluation of sports tourism, it can be regarded as a MCDM issue. This study adopts aggregation operators to process evaluation information because of their simplicity and understandability. Specifically, the Heronian mean operators are modified in linguistic neutrosophic environments to reflect the relations among multiple dimensions by adjusting two independence parameters. Furthermore, the Einstein operations of LNNs are defined to improve the flexibility of the aggregation operators-based method.

Considering the aforementioned motivations, this study aims to investigate the sustainability evaluation problems of sports tourism in linguistic neutrosophic environments. The main objectives are: First, the assessment of sustainability in sports tourism is often characterized by ambiguous evaluation data. They are processed and denoted with LNNs. Second, multiple decision-making indexes are identified and a sound evaluation index system can be constructed. Third, the interrelations among arguments are reflected by new linguistic neutrosophic aggregation operators. Last, a novel decision-making framework is established to solve sustainability evaluation problems of sports tourism.

The key novelty and contributions are as follows:

1. With respect to the intricacy of sustainability evaluation and the habits of human beings, LNNs are suggested to describe fuzzy criteria information. In this way, different attitudes and opinions of decision makers can be simultaneously reflected by three linguistic membership functions. It theoretically enhances the expression of vague decision-making information.
2. New evaluation criteria (institution and technology) are recognized, which constitute a complete sustainability evaluation criteria system of sports tourism together with the original three indexes (economy, sociality and environment). In this case, more respects can be considered and reflected by decision makers in the evaluation process, which makes practical contribution to sustainable sports tourism assessment.
3. New aggregation operators of LNNs, including the linguistic neutrosophic Einstein Heronian mean (LNEHM) operators and the weighted linguistic neutrosophic Einstein Heronian mean (WLNEHM) operators, are proposed. They are utilized to aggregate evaluation fuzzy evaluation information, which enriches the disposition of fuzzy information in theory. The main advantages of these operators are two-fold. On the one hand, they can capture the interactions among dependent indexes based on the Heronian mean operators; On the other hand, desired flexibility and robustness can be obtained with Einstein operations during the information fusion process.
4. A linguistic neutrosophic decision-making framework based on the aforementioned operators is built to assess the developmental performance of sustainable sports tourism under five evaluation dimensions. Furthermore, the superiority of the proposed method is verified through comparative and sensitivity analyses.

The organization of this research is as follows: Related literature is reviewed and discussed in “Literature review” Section. Some basic knowledge about LNNs is introduced in “Methods and materials” Section. In “New linguistic neutrosophic aggregation operators” Section, two kinds of new aggregation operators are presented to aggregate LNNs based on the Einstein operations and Heronian mean operators. In “Methodology” Section, a linguistic neutrosophic decision-making method is presented based on the proposed operators. In “Case Study” Section, an example of sustainability evaluation of ice and snow sports attractions is illustrated to show the decision-making process of our method. In “Analyses and discussions” Section, comparative and sensitivity analyses are conducted to testify the feasibility and efficiency of the proposed approach. In addition, some managerial insights and implications are derived. Finally, some necessary conclusions are provided in “Conclusions” Section.

## Literature review

Plenty of researchers have investigated tourism with sustainability from different perspectives [7]. In recent years, Hall [8] discussed sustainable tourism development using a heterogeneous constructionism method based on managerial ecology; Rasoolimanesh et al. [9] summarized the tourism sustainability influencing indexes related to sustainable development purposes; Roxas et al. [10] constructed a 5-point tourism stakeholder model to describe tourism stakeholders’ roles and collaborative merits in developing sustainable tourism destinations; Streimikiene et al. [11] explained the meaning and major challenges of sustainable tourism development from the competitiveness perspective; Mondal and Samaddar [12] reviewed responsible tourism with sustainable development and pointed out the future investigation direction; Grilli et al. [13] explored the prospective tourist preferences for sustainable tourism development in Small Island Developing States; and Destek and Aydın [14] analyzed the impact of tourism on sustainable development through empirical research.

Some researchers have explored sports tourism with sustainable development [15]. Recently, Hinch and Ito [16] discussed the sustainable sport tourism development situations in Japan; Chersulich Tomino et al. [17] analyzed the social impacts of outdoor sport-tourism events and made strategic event sustainability planning; Jiménez-García et al. [18] conducted a bibliometric analysis of sports tourism and sustainability from 2002 to 2019; Mascarenhas et al. [19] reviewed the environmental sustainability in the sports tourism market, while González-García et al. [20] investigated the impact of sports tourism on sustainable social development. However, only limited investigation has been made on concrete sport tourism sustainability evaluation approaches. Yang et al. [21] proposed the concept of sustainable sports tourism and evaluated sports tourism sustainability with four dimensions (economic, socio-cultural, environmental and institutional sustainability), but the technical element is ignored; and Yang et al. [22] discussed the relationships of sustainable sports tourism evaluation criteria with a two-stage decision-making framework. MCDM techniques are powerful tool to dispose evaluation problems with multiple evaluation dimensions [23, 24]. This study aims to propose a novel MCDM framework to assess sustainable sports tourism developmental performance in a linguistic neutrosophic environment.

The notion of linguistic term sets was put forward to depict these linguistic phrases [25]. Since then, many linguistic extensions have been emerged, such as hesitant fuzzy linguistic term sets [26], probabilistic linguistic term sets [27] and intuitionistic 2-tuple fuzzy linguistic numbers [28]. Moreover, decision making methods have been modified in different linguistic contexts to solve various evaluation issues, such as the appraisal of data platform vendors [29], the evaluation of tourist services [30], the expertise-based bid assessment for construction-contractor selection [31] and the evaluation of ship’s green degree [32]. Among them, linguistic intuitionistic fuzzy numbers (LIFNs) are proposed to simultaneously convey the membership degree and non-membership degree with linguistic terms [33]. Let $T=\{t_{0}=very\;low,\;t_{1}=low,\;t_{2}=alittle\;low,\;t_{3}=medium,\;t_{4}=a\;little\;high,\;t_{5}=high,\;t_{6}=very\;high\}$ be a linguistic term set. Take *lt* = [*t*_4_,*t*_1_] as an example, it shows that the membership degree is a little high while the non-membership degree is low. Nevertheless, the hesitant degree of decision makers is not considered in LIFNs. To overcome this limitation, LNNs with three linguistic membership degrees is defined by Fang and Ye [34]. Take *η* = (*t*_5_,*t*_3_,*t*_1_) as an example, it represents that the linguistic truth membership degree is high, the linguistic hesitancy membership degree is medium, and the linguistic false membership degree is low. A distinct superiority of LNNs is that it can contain the linguistic truth, hesitancy and false membership degrees at the same time.

Since then, many researchers began to introduce LNNs into fuzzy evaluation problems and proposed some linguistic neutrosophic decision-making methods, as shown in **Table A1** of **S1 Appendix**. Recently, Li et al. [35] proposed an evaluation based on distance from average solution (EDAS) approach under linguistic neutrosophic situations to choose ideal property management companies; Garg [36] put forward new algorithms based on the complex proportional assessment (COPRAS) approach and aggregation operators under linguistic neutrosophic circumstance; Liu and You [37] developed a new group decision-making method based on the bidirectional projection measure of LNNs; Wang et al. [38] extended the Visekriterijumska Optimizacija I Kom-promisno Resenje (VIKOR) method in linguistic neutrosophic contexts; Zhu et al. [39] established a comprehensive analyses model by combining LNNs, regret theory and the preference ranking organization method for enrichment evaluation (PROMETHEE) approach to assess failure modes risk priority; Bhaumik et al. [40] built a multi-objective matrix game model with LNNs to solve tourism management problems; and Zhang et al. [41] proposed a new distance measure-based group decision-making method in linguistic neutrosophic environments.

Moreover, aggregation operators are another efficient way to integrate evaluation information [42–44]. Different kinds of aggregation operators have been explored in linguistic neutrosophic circumstances. For example, Fang and Ye [34] defined the linguistic neutrosophic weighted arithmetic averaging (LNWAA) operator and linguistic neutrosophic weighted geometric averaging (LNWGA) operator; Liu and You [45] studied some linguistic neutrosophic Hamy mean operators; Fan et al. [46] put forward the linguistic neutrosophic normalized weighted Bonferroni mean (LNNWBM) operator and linguistic neutrosophic normalized weighted geometric Bonferroni mean (LNNWGBM) operator; Liang et al. [47] came up with the idea of the linguistic neutrosophic improved weighted Heronian mean (LNIWHM) operator and linguistic neutrosophic improved geometric weighted Heronian mean (LNIGWHM) operator; Luo et al. [48] developed the linguistic neutrosophic Maclaurin symmetric mean (LNMSM) operators to cope with performance evaluation of human resources; and Li et al. [49] considered the weight Muirhead Mean operators to allocate reliability indicators in engineering fields. Among them, both the Bonferroni mean and Heronian mean operators can convey the correlations of evaluation indexes [50]. This is a desired nature, especially for the performance evaluation problems. Many decision-making criteria, such as “knowledge/skill” and “innovation/ability”, usually have associations with each other. However, the interactions between two different inputs are calculated two times in the Bonferroni mean operators, which lead to redundant calculations. Compared with Bonferroni mean operators, Heronian mean operators also consider the interrelationships of a certain input and itself, which makes it more reasonable and effective.

However, most of these aggregation operators are based on the algebraic operations of LNNs, which is short of elasticity and robustness. As another kind of Archimedes t-conorm and t-norm, Einstein operations are more flexible and have good smooth features [51, 52]. Therefore, plenty of scholars utilized Einstein t-norm and t-conorm to model the union and intersection on various fuzzy numbers. For example, Fan et al. [53] established the Einstein sum, product, and exponentiation operational rules of LNNs; Riaz et al. [54] put forward the q-rung orthopair fuzzy Einstein aggregation operations to manage sustainable energy planning decisions; Sarkar and Biswas [55] explored the Einstein t-norms and t-conorms for linguistic Pythagorean fuzzy numbers; Kamacı et al. [56] defined the interval-valued picture hesitant fuzzy Einstein operational laws; Iampan et al. [57] presented several linear Diophantine fuzzy Einstein aggregation operators to deal with linear Diophantine fuzzy information; Ashraf et al. [58] proposed the hesitant fuzzy rough Einstein aggregation operators to solve site selection issue of wind power plants; and Janani et al. [59] aggregated complex Pythagorean fuzzy information based on Einstein operations for picking out the best breed of Horsegram.

In this study, the MCDM method based on aggregation operators is extended in a linguistic neutrosophic context to evaluate the developmental performance of sustainable sports tourism under five dimensions. The Einstein operations of LNNs are defined and new linguistic neutrosophic aggregation operators are proposed. Compared with other aggregation operators, a distinct advantage of our approach is that the correlations of different evaluation dimensions can be reflected without redundant calculations. Furthermore, the proposed LNEHM operators have much elasticity and robustness.

## Methods and materials

In this section, the definition, operational rules and preference relations of LNNs are introduced.

**Definition 1.** [34] Suppose $T=\{t_{i}|i\in [0,v]\}$ is a linguistic term set, then *η* = (*t*_*a*_,*t*_*b*_,*t*_*c*_) is called a LNN, where *t*_*a*_,*t*_*b*_,*t*_*c*_∈*T*, and *t*_*a*_, *t*_*b*_ and *t*_*c*_ is the linguistic truth, hesitancy and false membership degree, respectively.

**Definition 2.** [34] If *η* = (*t*_*a*_,*t*_*b*_,*t*_*c*_), $\eta_{1}=(t_{a_{1}},t_{b_{1}},t_{c_{1}})$ and $\eta_{2}=(t_{a_{2}},t_{b_{2}},t_{c_{2}})$ are LNNs, and *δ*>0, then their algebraic operational rules are

$$\eta_{1}{⊕}_{A}\eta_{2}=(t_{a_{1}},t_{b_{1}},t_{c_{1}}){⊕}_{A}(t_{a_{2}},t_{b_{2}},t_{c_{2}})=(t_{a_{1}+a_{2}-\frac{a_{1}a_{2}}{v}},t_{\frac{b_{1}b_{2}}{v}},t_{\frac{c_{1}c_{2}}{v}});$$ (1)

$$\eta_{1}{⊗}_{A}\eta_{2}=(t_{a_{1}},t_{b_{1}},t_{c_{1}}){⊗}_{A}(t_{a_{2}},t_{b_{2}},t_{c_{2}})=(t_{\frac{a_{1}a_{2}}{v}},t_{b_{1}+b_{2}-\frac{b_{1}b_{2}}{v}},t_{c_{1}+c_{2}-\frac{c_{1}c_{2}}{v}});$$ (2)

$$\delta \eta =\delta (t_{a},t_{b},t_{c})=(t_{v-v{\left(1-\frac{a}{v}\right)}^{\delta }},t_{v{\left(\frac{b}{v}\right)}^{\delta }},t_{v{\left(\frac{c}{v}\right)}^{\delta }});$$ (3)

$$\eta^{\delta }={\left(t_{a},t_{b},t_{c}\right)}^{\delta }=(t_{v{\left(\frac{a}{v}\right)}^{\delta }},t_{v-v{\left(1-\frac{b}{v}\right)}^{\delta }},t_{v-v{\left(1-\frac{c}{v}\right)}^{\delta }}).$$ (4)

**Example 1.** Given a linguistic term set

$T=\{t_{0}=very\;low,\;t_{1}=low,\;t_{2}=a\;little\;low,\;t_{3}=medium,\;t_{4}=a\;little\;high,\;t_{5}=high,\;t_{6}=very\;high\}$, *δ* = 2 and three LNNs *η* = (*t*_1_,*t*_2_,*t*_3_), *η*_1_ = (*t*_4_,*t*_3_,*t*_6_) and *η*_2_ = (*t*_3_,*t*_2_,*t*_1_). Based on **Definition 2**, $\eta_{1}{⊕}_{A}\eta_{2}=(t_{4},t_{3},t_{6}){⊕}_{A}(t_{3},t_{2},t_{1})=(t_{4+3-\frac{4\times 3}{6}},t_{\frac{3\times 2}{6}},t_{\frac{6\times 1}{6}})=(t_{5},t_{1},t_{1})$, $\eta_{1}{⊗}_{A}\eta_{2}=(t_{4},t_{3},t_{6}){⊗}_{A}(t_{3},t_{2},t_{1})=(t_{\frac{4\times 3}{6}},t_{3+2-\frac{3\times 2}{6}},t_{6+1-\frac{6\times 1}{6}})=(t_{2},t_{4},t_{6})$, $2\times \eta =2\times (t_{1},t_{2},t_{3})=(t_{6-6\times {\left(1-\frac{1}{6}\right)}^{2}},t_{6\times {\left(\frac{2}{6}\right)}^{2}},t_{6\times {\left(\frac{3}{6}\right)}^{2}})=(t_{11/6},t_{2/3},t_{3/2})$ and $\eta^{2}={\left(t_{1},t_{2},t_{3}\right)}^{2}=(t_{6\times {\left(\frac{1}{6}\right)}^{2}},t_{6-6\times {\left(1-\frac{2}{6}\right)}^{2}},t_{6-6\times {\left(1-\frac{3}{6}\right)}^{2}})=(t_{1/6},t_{10/3},t_{9/2})$.

Furthermore, Fan et al. [53] defined the following Einstein operations of LNNs to show better smoothness.

**Definition 3.** [53] Let *η* = (*t*_*a*_,*t*_*b*_,*t*_*c*_), $\eta_{1}=(t_{a_{1}},t_{b_{1}},t_{c_{1}})$ and $\eta_{2}=(t_{a_{2}},t_{b_{2}},t_{c_{2}})$ be LNNs, $\alpha =\frac{a}{v}$, $\beta =\frac{b}{v}$, $\gamma =\frac{c}{v}$, $\alpha_{1}=\frac{a_{1}}{v}$, $\beta_{1}=\frac{b_{1}}{v}$, $\gamma_{1}=\frac{c_{1}}{v}$, $\alpha_{2}=\frac{a_{2}}{v}$, $\beta_{2}=\frac{b_{2}}{v}$, $\gamma_{2}=\frac{c_{2}}{v}$ and *τ*>0, then the Einstein operational laws of LNNs are

$$\eta_{1}{⊕}_{E}\eta_{2}=(t_{\frac{v(\alpha_{1}+\alpha_{2})}{1+\alpha_{1}\alpha_{2}}},t_{\frac{v\beta_{1}\beta_{2}}{1+(1-\beta_{1})(1-\beta_{2})}},t_{\frac{v\gamma_{1}\gamma_{2}}{1+(1-\gamma_{1})(1-\gamma_{2})}}),$$ (5)

$$\eta_{1}{⊗}_{E}\eta_{2}=(t_{\frac{v\alpha_{1}\alpha_{2}}{1+(1-\alpha_{1})(1-\alpha_{2})}},t_{\frac{v(\beta_{1}+\beta_{2})}{1+\beta_{1}\beta_{2}}},t_{\frac{v(\gamma_{1}+\gamma_{2})}{1+\gamma_{1}\gamma_{2}}}),$$ (6)

$$\tau \eta =\tau (t_{a},t_{b},t_{c})=(t_{v\cdot \frac{{\left(1+\alpha \right)}^{\tau }-{\left(1-\alpha \right)}^{\tau }}{{\left(1+\alpha \right)}^{\tau }+{\left(1-\alpha \right)}^{\tau }}},t_{\frac{2v\beta^{\tau }}{{\left(2-\beta \right)}^{\tau }+\beta^{\tau }}},t_{\frac{2v\gamma^{\tau }}{{\left(2-\gamma \right)}^{\tau }+\gamma^{\tau }}}),$$ (7)

$$\eta^{\tau }={\left(t_{a},t_{b},t_{c}\right)}^{\tau }=(t_{\frac{2v\alpha^{\tau }}{{\left(2-\alpha \right)}^{\tau }+\alpha^{\tau }}},t_{v\cdot \frac{{\left(1+\beta \right)}^{\tau }-{\left(1-\beta \right)}^{\tau }}{{\left(1+\beta \right)}^{\tau }+{\left(1-\beta \right)}^{\tau }}},t_{v\cdot \frac{{\left(1+\gamma \right)}^{\tau }-{\left(1-\gamma \right)}^{\tau }}{{\left(1+\gamma \right)}^{\tau }+{\left(1-\gamma \right)}^{\tau }}}).$$ (8)

**Example 2.** If *v* = 6, *τ* = 2 and there are three LNNs *η* = (*t*_1_,*t*_2_,*t*_3_), *η*_1_ = (*t*_4_,*t*_3_,*t*_6_) and *η*_2_ = (*t*_3_,*t*_2_,*t*_1_), then, based on **Definition 3**, $\eta_{1}{⊕}_{E}\eta_{2}=(t_{4},t_{3},t_{6}){⊕}_{E}(t_{3},t_{2},t_{1})=(t_{21/4},t_{3/4},t_{1})$, $\eta_{1}{⊗}_{E}\eta_{2}=(t_{4},t_{3},t_{6}){⊗}_{E}(t_{3},t_{2},t_{1})=(t_{12/7},t_{30/7},t_{6})$, $2\times \eta =2\times (t_{1},t_{2},t_{3})=(t_{72/37},t_{6/13},t_{6/5})$ and $\eta^{2}={\left(t_{1},t_{2},t_{3}\right)}^{2}=(t_{6/61},t_{18/5},t_{24/5})$.

**Theorem 1.** [53] Given three LNNs *η* = (*t*_*a*_,*t*_*b*_,*t*_*c*_), $\eta_{1}=(t_{a_{1}},t_{b_{1}},t_{c_{1}})$ and $\eta_{2}=(t_{a_{2}},t_{b_{2}},t_{c_{2}})$, then some properties are satisfied as follows:

$$\eta_{1}{⊕}_{E}\eta_{2}=\eta_{2}{⊕}_{E}\eta_{1},$$ (9)

$$\eta_{1}{⊗}_{E}\eta_{2}=\eta_{2}{⊗}_{E}\eta_{1},$$ (10)

$$\eta {⊕}_{E}(\eta_{1}{⊕}_{E}\eta_{2})=(\eta {⊕}_{E}\eta_{1}){⊕}_{E}\eta_{2},$$ (11)

$$\eta {⊗}_{E}(\eta_{1}{⊗}_{E}\eta_{2})=(\eta {⊗}_{E}\eta_{1}){⊗}_{E}\eta_{2},$$ (12)

$$\tau (\eta_{1}{⊕}_{E}\eta_{2})=\tau \eta_{1}{⊕}_{E}\tau \eta_{2}\;(\tau >0),$$ (13)

$${\left(\eta_{1}{⊗}_{E}\eta_{2}\right)}^{\tau }={\eta_{1}}^{\tau }{⊗}_{E}{\eta_{2}}^{\tau }\;(\tau >0),$$ (14)

$$\tau_{1}\eta {⊕}_{E}\tau_{2}\eta =(\tau_{1}+\tau_{2})\eta \;(\tau_{1},\tau_{2}>0),$$ (15)

$$\eta^{\tau_{1}}{⊗}_{E}\eta^{\tau_{2}}=\eta^{\left(\tau_{1}+\tau_{2}\right)}\;(\tau_{1},\tau_{2}>0),$$ (16)

$$\tau_{1}(\tau_{2}\eta )=\tau_{1}\tau_{2}\eta \;(\tau_{1},\tau_{2}>0),$$ (17)

$${\left(\eta^{\tau_{1}}\right)}^{\tau_{2}}=\eta^{\tau_{1}\tau_{2}},\;(\tau_{1},\tau_{2}>0).$$ (18)

**Definition 4.** [34] Given a LNN *η* = (*t*_*a*_,*t*_*b*_,*t*_*c*_), then its score function *S*(*η*) and accuracy function *A*(*η*) are:

$$S(\eta )=\frac{2v+a-b-c}{3v},$$ (19)

$$A(\eta )=\frac{a-c}{v}.$$ (20)

**Example 3.** Suppose *v* = 6, *η*_1_ = (*t*_4_,*t*_3_,*t*_6_), *η*_2_ = (*t*_3_,*t*_2_,*t*_1_) and *η*_3_ = (*t*_2_,*t*_2_,*t*_5_) are LNNs, then, based on **Definition 4**, $S(\eta_{1})=\frac{2\times 6+4-3-6}{3\times 6}=\frac{7}{18}$, $S(\eta_{2})=\frac{2\times 6+3-2-1}{3\times 6}=\frac{2}{3}$, $S(\eta_{3})=\frac{2\times 6+2-2-5}{3\times 6}=\frac{7}{18}$, $A(\eta_{1})=\frac{4-6}{6}=-\frac{1}{3}$, $A(\eta_{2})=\frac{3-1}{6}=\frac{1}{3}$ and $A(\eta_{3})=\frac{2-5}{6}=-\frac{1}{2}$.

**Definition 5.** [34] Assume $\eta_{1}=(t_{a_{1}},t_{b_{1}},t_{c_{1}})$ and $\eta_{2}=(t_{a_{2}},t_{b_{2}},t_{c_{2}})$ are two LNNs, then

1. If *S*(*η*_1_)>*S*(*η*_2_), then *η*_1_≻*η*_2_;
2. If *S*(*η*_1_) = *S*(*η*_2_) and $\{\begin{array}{c} A(\eta_{1})>A(\eta_{2}),\;then\;\eta_{1}≻\eta_{2}\\ A(\eta_{1})=A(\eta_{2}),\;then\;\eta_{1}∼\eta_{2} \end{array}$.

**Example 4.** Suppose there are three LNNs *η*_1_ = (*t*_4_,*t*_3_,*t*_6_), *η*_2_ = (*t*_3_,*t*_2_,*t*_1_) and *η*_3_ = (*t*_2_,*t*_2_,*t*_5_), which are the same with **Example 3**. Based on **Definition 5**, because $S(\eta_{1})=\frac{7}{18}$, $S(\eta_{2})=\frac{2}{3}$ and *S*(*η*_2_)>*S*(*η*_1_), then *η*_2_≻*η*_1_; because $S(\eta_{2})=\frac{2}{3}$, $S(\eta_{3})=\frac{7}{18}$ and *S*(*η*_2_)>*S*(*η*_3_) then *η*_2_≻*η*_3_; because $S(\eta_{1})=S(\eta_{3})=\frac{7}{18}$ and $A(\eta_{1})>A(\eta_{3})$, then *η*_1_≻*η*_3_; because *η*_2_≻*η*_1_, *η*_2_≻*η*_3_ and *η*_1_≻*η*_3_, then *η*_2_≻*η*_1_≻*η*_3_.

## New linguistic neutrosophic aggregation operators

Compared with other aggregation operators, Einstein operations have better flexibility and smoothness, while Heronian mean operators are powerful tool to capture interrelationships among inputs. Hence, new aggregation operators of LNNs based on Einstein and Heronian mean operations are presented, and some special cases are discussed in this section.

### Linguistic neutrosophic Einstein Heronian mean operators

**Definition 6.** Suppose $\eta_{i}=(t_{a_{i}},t_{b_{i}},t_{c_{i}})$ (*i* = 1,2,…,*u*) is a group of LNNs, *x*≥0 and *y*≥0 are two independent parameters to reflect the interactions of criteria, then the LNEHM operator is

$$LNEHM^{x,y}(\eta_{1},\eta_{2},\cdots ,\eta_{u})={\left(\frac{2}{u(u+1)}\sum_{i=1,j=i}^{u}\left({\eta_{i}}^{x}{⊗}_{E}{\eta_{j}}^{y}\right)\right)}^{\frac{1}{x+y}}.$$ (21)

**Example 5.** Given a group of LNNs *η*_11_ = (*t*_1_,*t*_1_,*t*_4_), *η*_12_ = (*t*_4_,*t*_1_,*t*_2_), *η*_13_ = (*t*_4_,*t*_2_,*t*_1_) and *η*_14_ = (*t*_3_,*t*_3_,*t*_2_), let *x* = *y* = 1, then based on Eq (21), we have *u* = 4 and $LNEHM^{1,1}(\eta_{1},\eta_{2},\eta_{3},\eta_{4})={\left(\frac{2}{4\times (4+1)}\sum_{i=1,j=i}^{4}\left(\eta_{i}{⊗}_{E}\eta_{j}\right)\right)}^{\frac{1}{1+1}}=(t_{3.1627},t_{1.6439},t_{2.0986})$.

**Theorem 2.** Assume $\eta_{i}=(t_{a_{i}},t_{b_{i}},t_{c_{i}})$ (*i* = 1,2,…,*u*) is a group of LNNs and *x*,*y*≥0, let $\alpha_{i}=\frac{a_{i}}{v}$, $\beta_{i}=\frac{b_{i}}{v}$, $\gamma_{i}=\frac{c_{i}}{v}$, $\alpha_{j}=\frac{a_{j}}{v}$, $\beta_{j}=\frac{b_{j}}{v}$ and $\gamma_{j}=\frac{c_{j}}{v}$, then the aggregation result of the LNEHM operator is still a LNN, and have

$$LNEHM^{x,y}(\eta_{1},\eta_{2},\cdots ,\eta_{u})={\left(\frac{2}{u(u+1)}\sum_{i=1,j=i}^{u}\left({\eta_{i}}^{x}{⊗}_{E}{\eta_{j}}^{y}\right)\right)}^{\frac{1}{x+y}}=(t_{\frac{2v}{1+{\left(\frac{1+3\alpha }{1-\alpha }\right)}^{\frac{1}{x+y}}}},t_{v\cdot \frac{1-{\left(\frac{\beta -1}{\beta +3}\right)}^{\frac{1}{x+y}}}{1+{\left(\frac{\beta -1}{\beta +3}\right)}^{\frac{1}{x+y}}}},t_{v\cdot \frac{1-{\left(\frac{\gamma -1}{\gamma +3}\right)}^{\frac{1}{x+y}}}{1+{\left(\frac{\gamma -1}{\gamma +3}\right)}^{\frac{1}{x+y}}}}),$$ (22)

where $\alpha ={\left(\prod_{i=1,j=i}^{u}\frac{{\left(2-\alpha_{i}\right)}^{x}{\left(2-\alpha_{j}\right)}^{y}-{\alpha_{i}}^{x}{\alpha_{j}}^{y}}{{\left(2-\alpha_{i}\right)}^{x}{\left(2-\alpha_{j}\right)}^{y}+3{\alpha_{i}}^{x}{\alpha_{j}}^{y}}\right)}^{\frac{2}{u(u+1)}}$, $\beta ={\left(\prod_{i=1,j=i}^{u}\frac{{\left(1+\beta_{i}\right)}^{x}{\left(1+\beta_{j}\right)}^{y}+3{\left(1-\beta_{i}\right)}^{x}{\left(1-\beta_{j}\right)}^{y}}{{\left(1+\beta_{i}\right)}^{x}{\left(1+\beta_{j}\right)}^{y}-{\left(1-\beta_{i}\right)}^{x}{\left(1-\beta_{j}\right)}^{y}}\right)}^{\frac{2}{u(u+1)}}$ and $\gamma ={\left(\prod_{i=1,j=i}^{u}\frac{{\left(1+\gamma_{i}\right)}^{x}{\left(1+\gamma_{j}\right)}^{y}+3{\left(1-\gamma_{i}\right)}^{x}{\left(1-\gamma_{j}\right)}^{y}}{{\left(1+\gamma_{i}\right)}^{x}{\left(1+\gamma_{j}\right)}^{y}-{\left(1-\gamma_{i}\right)}^{x}{\left(1-\gamma_{j}\right)}^{y}}\right)}^{\frac{2}{u(u+1)}}$.

The proof of **Theorem 2** can be seen in **S2 Appendix**.

**Property 1.** (Idempotency) If $\eta_{1}=\eta_{2}=\cdots =\eta_{u}=\eta =(t_{a},t_{b},t_{c})$ and *x*,*y*≥0, then

$$LNEHM^{x,y}(\eta_{1},\eta_{2},\cdots ,\eta_{u})=\eta .$$ (23)

**Property 2.** (Permutation) Given a collection of LNNs $\eta_{i}=(t_{a_{i}},t_{b_{i}},t_{c_{i}})$ (*i* = 1,2,…,*u*), assume $\left({\eta_{1}}^{\prime },{\eta_{2}}^{\prime },\cdots ,{\eta_{u}}^{\prime }\right)$ is any permutation of (*η*_1_,*η*_2_,⋯,*η*_*u*_) and *x*,*y*≥0, then

$$LNEHM^{x,y}(\eta_{1},\eta_{2},\cdots ,\eta_{u})=LNEHM^{x,y}({\eta_{1}}^{\prime },{\eta_{2}}^{\prime },\cdots ,{\eta_{u}}^{\prime }).$$ (24)

**Property 3.** (Monotonicity) Suppose $\eta_{i}=(t_{a_{i}},t_{b_{i}},t_{c_{i}})$ and $\vartheta_{i}=(t_{d_{i}},t_{e_{i}},t_{f_{i}})$ are two collections of LNNs, *x*,*y*≥0, *a*_*i*_≥*d*_*i*_, *b*_*i*_≤*e*_*i*_ and *c*_*i*_≤*f*_*i*_ for all *i* = 1,2,…,*u*, then

$$LNEHM^{x,y}(\eta_{1},\eta_{2},\cdots ,\eta_{u})\geq LNEHM^{x,y}(\vartheta_{1},\vartheta_{2},\cdots ,\vartheta_{u}).$$ (25)

**Property 4.** (Boundness) Assume $\eta_{i}=(t_{a_{i}},t_{b_{i}},t_{c_{i}})$ (*i* = 1,2,…,*u*) is a collection of LNNs, *η*^−^ = min{*η*_1_,*η*_2_,⋯,*η*_*u*_}, *η*^+^ = max{*η*_1_,*η*_2_,⋯,*η*_*u*_} and *x*,*y*≥0, then

$$\eta^{-}\leq LNEHM^{x,y}(\eta_{1},\eta_{2},\cdots ,\eta_{u})\leq \eta^{+}.$$ (26)

When the LNEHM operators are allocated with diverse *x* and *y* values, special cases are acquired as follows:

**Special Case 1.** Suppose *x* = *y* = 1, then the LNEHM operator is degenerated into

$$LNEHM^{1,1}(\eta_{1},\eta_{2},\cdots ,\eta_{u})={\left(\frac{2}{u(u+1)}\sum_{i=1,j=i}^{u}\left(\eta_{i}{⊗}_{E}\eta_{j}\right)\right)}^{\frac{1}{2}}=(t_{\frac{2v}{1+{\left(\frac{1+3\alpha }{1-\alpha }\right)}^{\frac{1}{2}}}},t_{v\cdot \frac{1-{\left(\frac{\beta -1}{\beta +3}\right)}^{\frac{1}{2}}}{1+{\left(\frac{\beta -1}{\beta +3}\right)}^{\frac{1}{2}}}},t_{v\cdot \frac{1-{\left(\frac{\gamma -1}{\gamma +3}\right)}^{\frac{1}{2}}}{1+{\left(\frac{\gamma -1}{\gamma +3}\right)}^{\frac{1}{2}}}}),$$ (27)

where $\alpha ={\left(\prod_{i=1,j=i}^{u}\frac{2-\alpha_{i}-\alpha_{j}}{2-\alpha_{i}-\alpha_{j}+2\alpha_{i}\alpha_{j}}\right)}^{\frac{2}{u(u+1)}}$, $\beta ={\left(\prod_{i=1,j=i}^{u}\frac{2-\beta_{i}-\beta_{j}+2\beta_{i}\beta_{j}}{\beta_{i}+\beta_{j}}\right)}^{\frac{2}{u(u+1)}}$ and $\gamma ={\left(\prod_{i=1,j=i}^{u}\frac{2-\gamma_{i}-\gamma_{j}+2\gamma_{i}\gamma_{j}}{\gamma_{i}+\gamma_{j}}\right)}^{\frac{2}{u(u+1)}}$.

**Special Case 2.** Suppose *x* = 1 and *y*→0, then the LNEHM operator is degenerated into the linguistic neutrosophic Einstein averaging operator as follows:

$$LNEHM^{1,0}(\eta_{1},\eta_{2},\cdots ,\eta_{u})=\frac{1}{u}\sum_{i=1}^{u}\eta_{i}=(t_{v\cdot \frac{1-{\left(\prod_{i=1}^{u}\left(\frac{1-\alpha_{i}}{1+\alpha_{i}}\right)\right)}^{\frac{1}{u}}}{1+{\left(\prod_{i=1}^{u}\left(\frac{1-\alpha_{i}}{1+\alpha_{i}}\right)\right)}^{\frac{1}{u}}}},t_{\frac{2v}{1+{\left(\prod_{i=1}^{u}\left(\frac{2-\beta_{i}}{\beta_{i}}\right)\right)}^{\frac{1}{u}}}},t_{\frac{2v}{1+{\left(\prod_{i=1}^{u}\left(\frac{2-\gamma_{i}}{\gamma_{i}}\right)\right)}^{\frac{1}{u}}}}).$$ (28)

**Special Case 3.** Suppose *x*>0 and *y* = 0, then the LNEHM operator is degenerated into the linguistic neutrosophic Einstein generalized averaging operator as follows:

$$LNEHM^{x,0}(\eta_{1},\eta_{2},\cdots ,\eta_{u})={\left(\frac{1}{u}\sum_{i=1}^{u}{\eta_{i}}^{x}\right)}^{\frac{1}{x}}=(t_{\frac{2v}{1+{\left(\frac{1+3\alpha }{1-\alpha }\right)}^{\frac{1}{x}}}},t_{v\cdot \frac{1-{\left(\frac{\beta -1}{\beta +3}\right)}^{\frac{1}{x}}}{1+{\left(\frac{\beta -1}{\beta +3}\right)}^{\frac{1}{x}}}},t_{v\cdot \frac{1-{\left(\frac{\gamma -1}{\gamma +3}\right)}^{\frac{1}{x}}}{1+{\left(\frac{\gamma -1}{\gamma +3}\right)}^{\frac{1}{x}}}}),$$ (29)

where $\alpha ={\left(\prod_{i=1}^{u}\frac{{\left(2-\alpha_{i}\right)}^{x}-{\alpha_{i}}^{x}}{{\left(2-\alpha_{i}\right)}^{x}+3{\alpha_{i}}^{x}}\right)}^{\frac{1}{u}}$, $\beta ={\left(\prod_{i=1}^{u}\frac{{\left(1+\beta_{i}\right)}^{x}+3{\left(1-\beta_{i}\right)}^{x}}{{\left(1+\beta_{i}\right)}^{x}-{\left(1-\beta_{i}\right)}^{x}}\right)}^{\frac{1}{u}}$ and $\gamma ={\left(\prod_{i=1}^{u}\frac{{\left(1+\gamma_{i}\right)}^{x}+3{\left(1-\gamma_{i}\right)}^{x}}{{\left(1+\gamma_{i}\right)}^{x}-{\left(1-\gamma_{i}\right)}^{x}}\right)}^{\frac{1}{u}}$.

### Weighted linguistic neutrosophic Einstein Heronian mean operators

**Definition 7.** Let $\eta_{i}=(t_{a_{i}},t_{b_{i}},t_{c_{i}})$ (*i* = 1,2,…,*u*) be a collection of LNNs and *w* = (*w*_1_,*w*_2_,⋯,*w*_*u*_)^*T*^ be the weight vector of *η*_*i*_(*i* = 1,2,…,*u*), where 0≤*w*_1_,*w*_2_,⋯,*w*_*u*_≥1, *w*_1_+*w*_2_+⋯+*w*_*u*_ = 1 and *x*,*y*≥0, then the WLNEHM operator is

$$WLNEHM_{w}^{x,y}(\eta_{1},\eta_{2},\cdots ,\eta_{u})={\left(\frac{2}{u(u+1)}\sum_{i=1,j=i}^{u}{\left(w_{i}\eta_{i}\right)}^{x}{⊗}_{E}{\left(w_{j}\eta_{j}\right)}^{y}\right)}^{\frac{1}{x+y}}.$$ (30)

**Example 6.** Suppose *η*_21_ = (*t*_4_,*t*_2_,*t*_1_), *η*_22_ = (*t*_5_,*t*_1_,*t*_3_), *η*_23_ = (*t*_3_,*t*_4_,*t*_1_), *η*_24_ = (*t*_4_,*t*_1_,*t*_2_) and *η*_25_ = (*t*_5_,*t*_2_,*t*_3_) is set of LNNs, while the weight vector is $w={\left(w_{1},w_{2},w_{3},w_{4},w_{5}\right)}^{T}={\left(0.241,0.276,0.159,0.168,0.156\right)}^{T}$, and let *x* = *y* = 1, then based on Eq (30), we have *u* = 5 and $WLNEHM_{w}^{1,1}(\eta_{1},\eta_{2},\eta_{3},\eta_{4},\eta_{5})={\left(\frac{2}{5\times (5+1)}\sum_{i=1,j=i}^{5}\left(w_{i}\eta_{i}){⊗}_{E}(w_{j}\eta_{j}\right)\right)}^{\frac{1}{1+1}}=(t_{1.1369},t_{4.8869},t_{4.9654}).$ **Theorem 3.** Given a set of LNNs $\eta_{i}=(t_{a_{i}},t_{b_{i}},t_{c_{i}})$ (*i* = 1,2,…,*u*), let $\alpha_{i}=\frac{a_{i}}{v}$, $\beta_{i}=\frac{b_{i}}{v}$, $\gamma_{i}=\frac{c_{i}}{v}$, $\alpha_{j}=\frac{a_{j}}{v}$, $\beta_{j}=\frac{b_{j}}{v}$, $\gamma_{j}=\frac{c_{j}}{v}$ and *x*,*y*≥0, then the aggregation result of the WLNEHM operator is still a LNN, and have

$$WLNEHM_{w}^{x,y}(\eta_{1},\eta_{2},\cdots ,\eta_{u})={\left(\frac{2}{u(u+1)}\sum_{i=1,j=i}^{u}{\left(w_{i}\eta_{i}\right)}^{x}{⊗}_{E}{\left(w_{j}\eta_{j}\right)}^{y}\right)}^{\frac{1}{x+y}}=(t_{\frac{2v}{1+{\left(\frac{1+3\alpha }{1-\alpha }\right)}^{\frac{1}{x+y}}}},t_{v\cdot \frac{1-{\left(\frac{\beta -1}{\beta +3}\right)}^{\frac{1}{x+y}}}{1+{\left(\frac{\beta -1}{\beta +3}\right)}^{\frac{1}{x+y}}}},t_{v\cdot \frac{1-{\left(\frac{\gamma -1}{\gamma +3}\right)}^{\frac{1}{x+y}}}{1+{\left(\frac{\gamma -1}{\gamma +3}\right)}^{\frac{1}{x+y}}}}),$$ (31)

where $\alpha ={\left(\prod_{i=1,j=i}^{u}\frac{{m_{i}}^{x}{m_{j}}^{y}-1}{{m_{i}}^{x}{m_{j}}^{y}+3}\right)}^{\frac{2}{u(u+1)}}$, $\beta ={\left(\prod_{i=1,j=i}^{u}\frac{1+3{n_{i}}^{x}{n_{j}}^{y}}{1-{n_{i}}^{x}{n_{j}}^{y}}\right)}^{\frac{2}{u(u+1)}}$, $\gamma ={\left(\prod_{i=1,j=i}^{u}\frac{1+3{l_{i}}^{x}{l_{j}}^{y}}{1-{l_{i}}^{x}{l_{j}}^{y}}\right)}^{\frac{2}{u(u+1)}}$, $m_{i}=\frac{{\left(1+\alpha_{i}\right)}^{w_{i}}+3{\left(1-\alpha_{i}\right)}^{w_{i}}}{{\left(1+\alpha_{i}\right)}^{w_{i}}-{\left(1-\alpha_{i}\right)}^{w_{i}}}$, $n_{i}=\frac{{\left(2-\beta_{i}\right)}^{w_{i}}-{\beta_{i}}^{w_{i}}}{{\left(2-\beta_{i}\right)}^{w_{i}}+3{\beta_{i}}^{w_{i}}}$, $l_{i}=\frac{{\left(2-\gamma_{i}\right)}^{w_{i}}-{\gamma_{i}}^{w_{i}}}{{\left(2-\gamma_{i}\right)}^{w_{i}}+3{\gamma_{i}}^{w_{i}}}$, $m_{j}=\frac{{\left(1+\alpha_{j}\right)}^{w_{j}}+3{\left(1-\alpha_{j}\right)}^{w_{j}}}{{\left(1+\alpha_{j}\right)}^{w_{j}}-{\left(1-\alpha_{j}\right)}^{w_{j}}}$, $n_{j}=\frac{{\left(2-\beta_{j}\right)}^{w_{j}}-{\beta_{j}}^{w_{j}}}{{\left(2-\beta_{j}\right)}^{w_{j}}+3{\beta_{j}}^{w_{j}}}$ and $l_{j}=\frac{{\left(2-\gamma_{j}\right)}^{w_{j}}-{\gamma_{j}}^{w_{j}}}{{\left(2-\gamma_{j}\right)}^{w_{j}}+3{\gamma_{j}}^{w_{j}}}$.

Because the proof of **Theorem 3** is similar to that of **Theorem 2**, it is left out here.

**Property 5.** (Permutation) Suppose $\eta_{i}=(t_{a_{i}},t_{b_{i}},t_{c_{i}})$ (*i* = 1,2,…,*u*) is a group of LNNs, assume $\left({\eta_{1}}^{\prime },{\eta_{2}}^{\prime },\cdots ,{\eta_{u}}^{\prime }\right)$ is any permutation of (*η*_1_,*η*_2_,⋯,*η*_*u*_) and *x*,*y*≥0, then

$$WLNEHM_{w}^{x,y}(\eta_{1},\eta_{2},\cdots ,\eta_{u})=WLNEHM_{w}^{x,y}({\eta_{1}}^{\prime },{\eta_{2}}^{\prime },\cdots ,{\eta_{u}}^{\prime }).$$ (32)

**Property 6.** (Monotonicity) Let $\eta_{i}=(t_{a_{i}},t_{b_{i}},t_{c_{i}})$ and $\vartheta_{i}=(t_{d_{i}},t_{e_{i}},t_{f_{i}})$ be two sets of LNNs and *x*,*y*≥0, if *a*_*i*_≥*d*_*i*_, *b*_*i*_≤*e*_*i*_ and *c*_*i*_≤*f*_*i*_ for all *i* = 1,2,…,*u*, then

$$WLNEHM_{w}^{x,y}(\eta_{1},\eta_{2},\cdots ,\eta_{u})\geq WLNEHM_{w}^{x,y}(\vartheta_{1},\vartheta_{2},\cdots ,\vartheta_{u}).$$ (33)

**Property 7.** (Boundness) Assume $\eta_{i}=(t_{a_{i}},t_{b_{i}},t_{c_{i}})$ (*i* = 1,2,…,*u*) is a collection of LNNs, *η*^−^ = min{*η*_1_,*η*_2_,⋯,*η*_*u*_}, *η*^+^ = max{*η*_1_,*η*_2_,⋯,*η*_*u*_} and *x*,*y*≥0, then

$$\eta^{-}\leq WLNEHM_{w}^{x,y}(\eta_{1},\eta_{2},\cdots ,\eta_{u})\leq \eta^{+}.$$ (34)

When the WLNEHM operators are allocated with diverse *x* and *y* values, special cases are acquired as follows:

**Special Case 4.** Suppose *x* = *y* =1, then the WLNEHM operator is degenerated into

$$WLNEHM_{w}^{1,1}(\eta_{1},\eta_{2},\cdots ,\eta_{u})={\left(\frac{2}{u(u+1)}\sum_{i=1,j=i}^{u}\left(w_{i}\eta_{i}){⊗}_{E}(w_{j}\eta_{j}\right)\right)}^{\frac{1}{2}}=(t_{\frac{2v}{1+{\left(\frac{1+3\alpha }{1-\alpha }\right)}^{\frac{1}{2}}}},t_{v\cdot \frac{1-{\left(\frac{\beta -1}{\beta +3}\right)}^{\frac{1}{2}}}{1+{\left(\frac{\beta -1}{\beta +3}\right)}^{\frac{1}{2}}}},t_{v\cdot \frac{1-{\left(\frac{\gamma -1}{\gamma +3}\right)}^{\frac{1}{2}}}{1+{\left(\frac{\gamma -1}{\gamma +3}\right)}^{\frac{1}{2}}}}),$$ (35)

where $\alpha ={\left(\prod_{i=1,j=i}^{u}\left(\frac{(\frac{{\left(1+\alpha_{i}\right)}^{w_{i}}+3{\left(1-\alpha_{i}\right)}^{w_{i}}}{{\left(1+\alpha_{i}\right)}^{w_{i}}-{\left(1-\alpha_{i}\right)}^{w_{i}}})(\frac{{\left(1+\alpha_{j}\right)}^{w_{j}}+3{\left(1-\alpha_{j}\right)}^{w_{j}}}{{\left(1+\alpha_{j}\right)}^{w_{j}}-{\left(1-\alpha_{j}\right)}^{w_{j}}})-1}{(\frac{{\left(1+\alpha_{i}\right)}^{w_{i}}+3{\left(1-\alpha_{i}\right)}^{w_{i}}}{{\left(1+\alpha_{i}\right)}^{w_{i}}-{\left(1-\alpha_{i}\right)}^{w_{i}}})(\frac{{\left(1+\alpha_{j}\right)}^{w_{j}}+3{\left(1-\alpha_{j}\right)}^{w_{j}}}{{\left(1+\alpha_{j}\right)}^{w_{j}}-{\left(1-\alpha_{j}\right)}^{w_{j}}})+3}\right)\right)}^{\frac{2}{u(u+1)}}$, $\beta ={\left(\prod_{i=1,j=i}^{u}\left(\frac{1+3(\frac{{\left(2-\beta_{i}\right)}^{w_{i}}-{\beta_{i}}^{w_{i}}}{{\left(2-\beta_{i}\right)}^{w_{i}}+3{\beta_{i}}^{w_{i}}})(\frac{{\left(2-\beta_{j}\right)}^{w_{j}}-{\beta_{j}}^{w_{j}}}{{\left(2-\beta_{j}\right)}^{w_{j}}+3{\beta_{j}}^{w_{j}}})}{1-(\frac{{\left(2-\beta_{i}\right)}^{w_{i}}-{\beta_{i}}^{w_{i}}}{{\left(2-\beta_{i}\right)}^{w_{i}}+3{\beta_{i}}^{w_{i}}})(\frac{{\left(2-\beta_{j}\right)}^{w_{j}}-{\beta_{j}}^{w_{j}}}{{\left(2-\beta_{j}\right)}^{w_{j}}+3{\beta_{j}}^{w_{j}}})}\right)\right)}^{\frac{2}{u(u+1)}}$ and $\gamma ={\left(\prod_{i=1,j=i}^{u}\left(\frac{1+3(\frac{{\left(2-\gamma_{i}\right)}^{w_{i}}-{\gamma_{i}}^{w_{i}}}{{\left(2-\gamma_{i}\right)}^{w_{i}}+3{\gamma_{i}}^{w_{i}}})(\frac{{\left(2-\gamma_{j}\right)}^{w_{j}}-{\gamma_{j}}^{w_{j}}}{{\left(2-\gamma_{j}\right)}^{w_{j}}+3{\gamma_{j}}^{w_{j}}})}{1-(\frac{{\left(2-\gamma_{i}\right)}^{w_{i}}-{\gamma_{i}}^{w_{i}}}{{\left(2-\gamma_{i}\right)}^{w_{i}}+3{\gamma_{i}}^{w_{i}}})(\frac{{\left(2-\gamma_{j}\right)}^{w_{j}}-{\gamma_{j}}^{w_{j}}}{{\left(2-\gamma_{j}\right)}^{w_{j}}+3{\gamma_{j}}^{w_{j}}})}\right)\right)}^{\frac{2}{u(u+1)}}$.

**Special Case 5.** Suppose *x* = 1 and *y*→0, then the WLNEHM operator is degenerated into the linguistic neutrosophic Einstein weighted averaging operator as follows:

$$WLNEHM_{w}^{1,0}(\eta_{1},\eta_{2},\cdots ,\eta_{u})=\frac{1}{u}\sum_{i=1}^{u}w_{i}\eta_{i}=(t_{v\cdot \frac{1-{\left(\prod_{i=1}^{u}{\left(\frac{1-\alpha_{i}}{1+\alpha_{i}}\right)}^{w_{i}}\right)}^{\frac{1}{u}}}{1+{\left(\prod_{i=1}^{u}{\left(\frac{1-\alpha_{i}}{1+\alpha_{i}}\right)}^{w_{i}}\right)}^{\frac{1}{u}}}},t_{\frac{2v}{1+{\left(\prod_{i=1}^{u}{\left(\frac{2-\beta_{i}}{\beta_{i}}\right)}^{w_{i}}\right)}^{\frac{1}{u}}}},t_{\frac{2v}{1+{\left(\prod_{i=1}^{u}{\left(\frac{2-\gamma_{i}}{\gamma_{i}}\right)}^{w_{i}}\right)}^{\frac{1}{u}}}}).$$ (36)

**Special Case 6.** Suppose *x*>0 and *y* = 0, then the WLNEHM operator is degenerated into the linguistic neutrosophic Einstein generalized weighted averaging operator as follows:

$$WLNEHM_{w}^{x,0}(\eta_{1},\eta_{2},\cdots ,\eta_{u})={\left(\frac{1}{u}\sum_{i=1}^{u}{\left(w_{i}\eta_{i}\right)}^{x}\right)}^{\frac{1}{x}}=(t_{\frac{2v}{1+{\left(\frac{1+3\alpha }{1-\alpha }\right)}^{\frac{1}{x}}}},t_{v\cdot \frac{1-{\left(\frac{\beta -1}{\beta +3}\right)}^{\frac{1}{x}}}{1+{\left(\frac{\beta -1}{\beta +3}\right)}^{\frac{1}{x}}}},t_{v\cdot \frac{1-{\left(\frac{\gamma -1}{\gamma +3}\right)}^{\frac{1}{x}}}{1+{\left(\frac{\gamma -1}{\gamma +3}\right)}^{\frac{1}{x}}}}),$$ (37)

where $\alpha ={\left(\prod_{i=1}^{u}\left(\frac{{\left(\frac{{\left(1+\alpha_{i}\right)}^{w_{i}}+3{\left(1-\alpha_{i}\right)}^{w_{i}}}{{\left(1+\alpha_{i}\right)}^{w_{i}}-{\left(1-\alpha_{i}\right)}^{w_{i}}}\right)}^{x}-1}{{\left(\frac{{\left(1+\alpha_{i}\right)}^{w_{i}}+3{\left(1-\alpha_{i}\right)}^{w_{i}}}{{\left(1+\alpha_{i}\right)}^{w_{i}}-{\left(1-\alpha_{i}\right)}^{w_{i}}}\right)}^{x}+3}\right)\right)}^{\frac{1}{u}}$, $\begin{array}{l} \beta ={\left(\prod_{i=1}^{u}\left(\frac{1+3{\left(\frac{{\left(2-\beta_{i}\right)}^{w_{i}}-{\beta_{i}}^{w_{i}}}{{\left(2-\beta_{i}\right)}^{w_{i}}+3{\beta_{i}}^{w_{i}}}\right)}^{x}}{1-{\left(\frac{{\left(2-\beta_{i}\right)}^{w_{i}}-{\beta_{i}}^{w_{i}}}{{\left(2-\beta_{i}\right)}^{w_{i}}+3{\beta_{i}}^{w_{i}}}\right)}^{x}}\right)\right)}^{\frac{1}{u}} \end{array}$ and $\gamma ={\left(\prod_{i=1}^{u}\left(\frac{1+3{\left(\frac{{\left(2-\gamma_{i}\right)}^{w_{i}}-{\gamma_{i}}^{w_{i}}}{{\left(2-\gamma_{i}\right)}^{w_{i}}+3{\gamma_{i}}^{w_{i}}}\right)}^{x}}{1-{\left(\frac{{\left(2-\gamma_{i}\right)}^{w_{i}}-{\gamma_{i}}^{w_{i}}}{{\left(2-\gamma_{i}\right)}^{w_{i}}+3{\gamma_{i}}^{w_{i}}}\right)}^{x}}\right)\right)}^{\frac{1}{u}}$.

## Methodology

**Fig 1:**
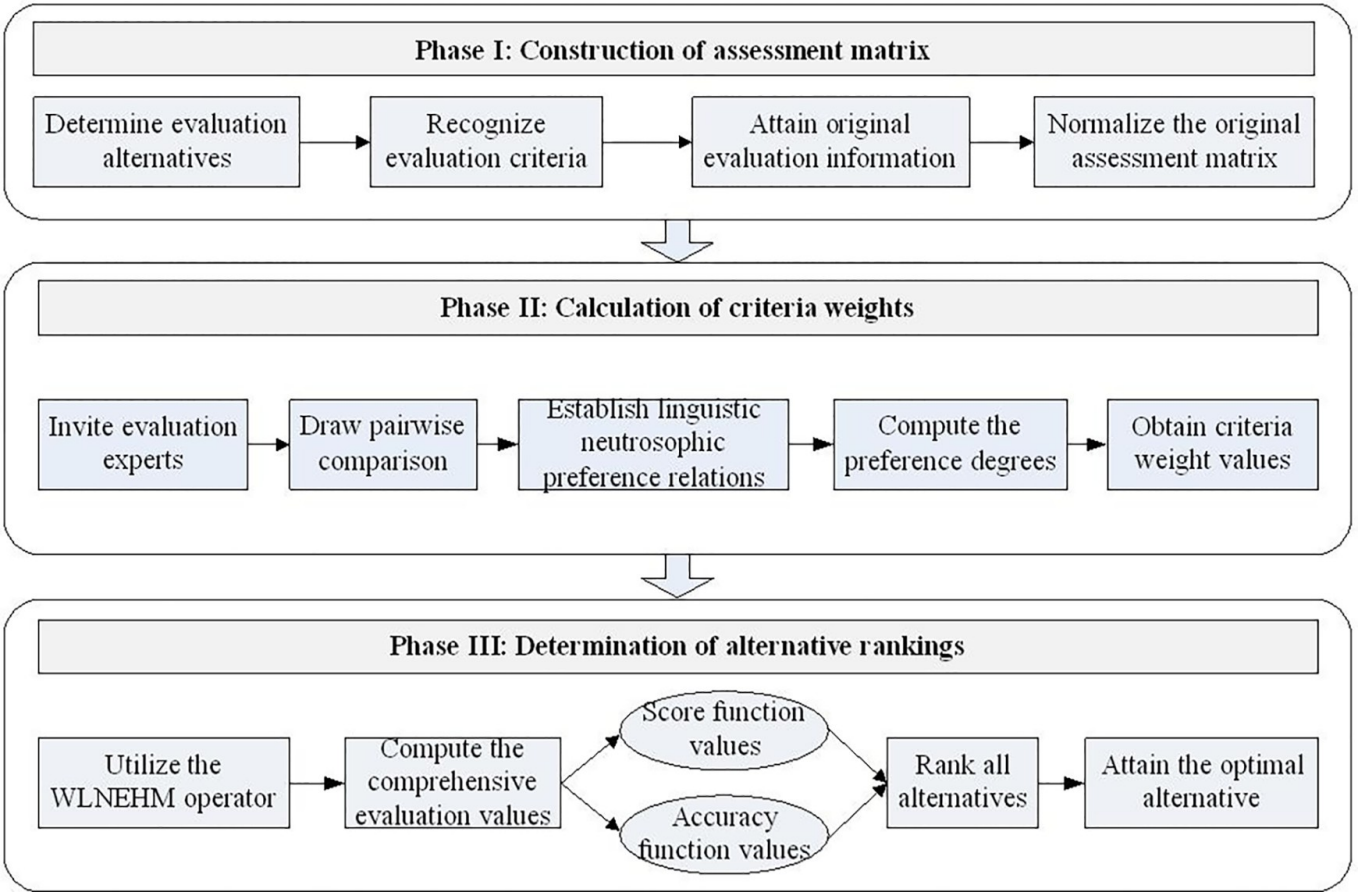
Framework of the proposed methodology.

In this section, a new decision-making approach is established with the proposed aggregation operators. The framework of the proposed methodology is shown in **Fig 1**.

### Phase I: Construction of assessment matrix

In this phase, evaluation information is obtained in a linguistic neutrosophic environment. Thereafter, a normalized decision-making matrix is established by standardizing the original assessment values.

**Step 1:** Attain original evaluation information.

Let {*P*_1_,*P*_2_,…,*P*_*s*_} be a set of alternatives and {*Q*_1_,*Q*_2_,…*Q*_*k*_} be a set of criteria. Considering the vagueness of human cognitions, evaluation results of each alternative *P*_*i*_(*i* = 1,2,…,*s*) under each criterion *Q*_*j*_(*j* = 1,2,…,*k*) are made by experts in the form of LNNs $\eta_{ij}=(t_{a_{ij}},t_{b_{ij}},t_{c_{ij}})$. Therefore, an assessment matrix is built in a linguistic neutrosophic environment, as follows:

$$N={\left(\eta_{ij}\right)}_{s\times k}=\begin{array}{cc} & \begin{array}{cccc} Q_{1} & Q_{2} & \cdots & Q_{k} \end{array}\\ \begin{array}{c} P_{1}\\ P_{2}\\ \vdots \\ P_{s} \end{array} & \left[\begin{array}{cccc} \eta_{11} & \eta_{12} & \cdots & \eta_{1k}\\ \eta_{21} & \eta_{22} & \cdots & \eta_{2k}\\ \vdots & \vdots & & \vdots \\ \eta_{s1} & \eta_{s2} & \cdots & \eta_{sk} \end{array}\right] \end{array}.$$ (38)

**Step 2:** Normalize the original assessment matrix.

When different types of criteria (benefit or cost criteria) are simultaneously in the original assessment matrix, they should be normalized with the following rules [34]:

$${\tilde{\eta }}_{ij}=\{\begin{array}{l} \;(t_{a_{ij}},t_{b_{ij}},t_{c_{ij}})\;for\;benefit\;criteria\\ (t_{v-a_{ij}},t_{v-b_{ij}},t_{v-c_{ij}})\;for\;\cos t\;criteria \end{array}.$$ (39)

Thus, a normalized decision-making matrix is obtained, and denoted as $\tilde{N}={\left({\tilde{\eta }}_{ij}\right)}_{s\times k}={\left({\tilde{t}}_{a_{ij}},{\tilde{t}}_{b_{ij}},{\tilde{t}}_{c_{ij}}\right)}_{s\times k}$.

### Phase II: Calculation of criteria weights

In this phase, the criteria weights are determined based on the linguistic neutrosophic preference relations and the proposed LNEHM operator.

**Step 3:** Establish linguistic neutrosophic preference relations.

When decision makers draw pairwise comparison among evaluation criteria using LNNs, linguistic neutrosophic preference relations *R* can be constructed as follows [25]:

$$R=[\begin{array}{ccccc} - & r_{12} & \cdots & r_{1(k-1)} & r_{1k}\\ r_{21} & - & & r_{2(k-1)} & r_{2k}\\ \vdots & \vdots & - & \vdots & \vdots \\ r_{(k-1)1} & r_{(k-1)2} & & - & r_{k(k-1)}\\ r_{k1} & r_{k2} & \cdots & r_{k(k-1)} & - \end{array}],$$ (40)

where $r_{js}=(t_{a_{rjs}},t_{b_{rjs}},t_{c_{rjs}})$, denoted as a LNN, represents the fuzzy preference of criterion *Q*_*j*_ (*j* = 1,2,⋯,*k*) for *Q*_*s*_ (*s* = 1,2,⋯,*k*,*s*≠*j*). Take *r*_12_ as an instance, it means the linguistic neutrosophic preference degree of criterion *Q*_1_ for *Q*_2_.

**Step 4:** Compute the overall preference degrees of criteria.

Based on the LNEHM operator presented in Section 4, the overall preference degrees of criteria can be computed with

$$r_{j}=LNEHM^{x,y}(r_{js})\;(s=1,2,\cdots ,k,s\neq j).$$ (41)

**Step 5:** Obtain the final criteria weight values.

The final criteria weight values can be determined with [47]

$$w_{j}=\frac{S(r_{j})}{\sum_{j=1}^{k}S(r_{j})}\;(j=1,2,\cdots ,k).$$ (42)

### Phase III: Determination of alternative rankings

After the evaluation matrix is normalized and the criteria weights are determined, a new evaluation approach with the presented WLNEHM operator is developed to rank alternatives with the following process.

**Step 6:** Compute the comprehensive evaluation values using the WLNEHM operator.

Based on the WLNEHM operator proposed in Section 4, the comprehensive evaluation values of alternatives can be calculated with

$$C_{i}=WLNEHM_{w}^{x,y}({\tilde{\eta }}_{i1},{\tilde{\eta }}_{i2},\cdots ,{\tilde{\eta }}_{ik}).$$ (43)

**Step 7:** Calculate the score function and accuracy function values.

The value of score function *S*(*C*_*i*_) and accuracy function *A*(*C*_*i*_) can be computed with Eqs (19) and (20).

**Step 8:** Attain the optimal alternative.

According to the result in Step 7, the rank order of alternatives can be derived. Namely, if the score function of two alternatives are unequal, a larger score function value means a better alternative; and if the score function of two alternatives are equal, a lager accuracy function value means a better alternative. When all alternatives are ranked, the optimal alternative can be easily selected.

**Ethics statement.** The submission does not require an ethics statement.

## Case study

In this section, a case of assessing the developmental performance of sustainable sports tourism is investigated.

### Background

As a kind of regional tourism activity, sports tourism has the basic attribute of sustainable development. Its goal is to promote the sustainable development of regional society, economy and environment. In essence, it reflects the pursuit of human beings for fair and coordinated development. Three consensual dimensions of sustainability (i.e., social, economic and environmental sustainability) are discussed in most tourism sustainability literature [7–14]. Additionally, some researchers concluded that institutional and technological support are also necessary elements in sustainable tourism evaluation [21, 22, 60–62]. Based on these literature [21, 22, 60–62], the sustainability evaluation criteria system of sports tourism is shown in **Table 1**.

**Table 1:** Sustainability evaluation criteria system of sports tourism.

Criterion	Type	Description
Economy *Q*_1_	Benefit	It is mainly reflected by local employment opportunity, sports diversity and economic feasibility promotion of local sports culture [60].
Sociality *Q*_2_	Benefit	It is mainly reflected by social equity and welfare, tourist service, emergency response and rescue, protection of residents’ basic rights and enrichment of local features [61].
Institution *Q*_3_	Benefit	It is mainly reflected by regional ordinance protection, policy promotion and marketing, sports tourism land planning and local government involvement [21].
Environment *Q*_4_	Benefit	It is mainly reflected by sports facility integrity, waste recycling, biodiversity and low environmental pollution [22].
Technology *Q*_5_	Benefit	It is mainly reflected by technology compatibility, the balance between old and new technologies (such as the environmental monitoring technology, information and communication technologies), and the usefulness, availability, efficiency, reliability, security and safety of these technologies in sports tourism [62].

### Illustrative example

In the northern cities of China, the ice and snow tourism industry has become one of the pillar industries. The successful holding of the 24^th^ Beijing Winter Olympics Games in 2022 brings great development opportunity for sustainable development of ice and snow sports tourism. Some local governments have actively established ice and snow sports tourism brands and adopted sustainable development strategies to promote the physical and mental health of the masses and drive local economic development. Given four ice and snow sports attractions {*P*_1_,*P*_2_,*P*_3_,*P*_4_}, the proposed method in Section 5 is adopted to assess their sustainability developmental performance.

In Phase I, the assessment matrix is constructed in a linguistic neutrosophic context. First, a structural questionnaire is designed with five dimensions (namely, economy, sociality, institution, environment and technology) based on **Table 1**. The sustainability degree of ice and snow sports attractions is measured with a 7-Likert scale, and the related linguistic term set is $T=\{t_{0}=very\;low,\;t_{1}=low,\;t_{2}=a\;little\;low,\;t_{3}=medium,\;t_{4}=a\;little\;high,\;t_{5}=high,\;t_{6}=very\;high\}$. Then, the field experts, scholars, government and practitioner representatives are organized to make assessments according to their knowledge and experience. In view of the unquantifiable nature of most criteria and diversity of decision makers’ opinions, LNNs are finally used to describe the evaluation results. As a result, an initial decision-making matrix can be built. In this research, an example of the assessment information represented with LNNs after questionnaire survey is shown in **Table 2**.

**Table 2:** Initial decision-making matrix *N*.

*N*	*Q*_1_	*Q*_2_	*Q*_3_	*Q*_4_	*Q*_5_
*P*_1_	(*t*_4_,*t*_2_,*t*_1_)	(*t*_4_,*t*_2_,*t*_1_)	(*t*_3_,*t*_4_,*t*_1_)	(*t*_4_,*t*_1_,*t*_2_)	(*t*_5_,*t*_2_,*t*_3_)
*P*_2_	(*t*_5_,*t*_1_,*t*_2_)	(*t*_5_,*t*_1_,*t*_1_)	(*t*_3_,*t*_1_,*t*_3_)	(*t*_3_,*t*_2_,*t*_3_)	(*t*_4_,*t*_3_,*t*_2_)
*P*_3_	(*t*_4_,*t*_3_,*t*_1_)	(*t*_5_,*t*_2_,*t*_3_)	(*t*_4_,*t*_4_,*t*_1_)	(*t*_3_,*t*_1_,*t*_2_)	(*t*_3_,*t*_2_,*t*_1_)
*P*_4_	(*t*_4_,*t*_1_,*t*_2_)	(*t*_4_,*t*_4_,*t*_1_)	(*t*_3_,*t*_1_,*t*_3_)	(*t*_4_,*t*_2_,*t*_3_)	(*t*_3_,*t*_2_,*t*_2_)

Because five evaluation criteria are all benefit criteria, they do not need to be normalized. Namely, the normalized decision-making matrix is the same as the initial matrix, as shown in **Table 3**.

**Table 3:** Normalized decision-making matrix $\tilde{N}$.

$\tilde{N}$	*Q*_1_	*Q*_2_	*Q*_3_	*Q*_4_	*Q*_5_
*P*_1_	(*t*_4_,*t*_2_,*t*_1_)	(*t*_5_,*t*_1_,*t*_3_)	(*t*_3_,*t*_4_,*t*_1_)	(*t*_4_,*t*_1_,*t*_2_)	(*t*_5_,*t*_2_,*t*_3_)
*P*_2_	(*t*_5_,*t*_1_,*t*_2_)	(*t*_5_,*t*_1_,*t*_1_)	(*t*_3_,*t*_1_,*t*_3_)	(*t*_3_,*t*_2_,*t*_3_)	(*t*_4_,*t*_3_,*t*_2_)
*P*_3_	(*t*_4_,*t*_3_,*t*_1_)	(*t*_5_,*t*_2_,*t*_3_)	(*t*_4_,*t*_4_,*t*_1_)	(*t*_3_,*t*_1_,*t*_2_)	(*t*_3_,*t*_2_,*t*_1_)
*P*_4_	(*t*_4_,*t*_1_,*t*_2_)	(*t*_4_,*t*_4_,*t*_1_)	(*t*_3_,*t*_1_,*t*_3_)	(*t*_4_,*t*_2_,*t*_3_)	(*t*_3_,*t*_2_,*t*_2_)

In Phase II, the criteria weights are calculated. First, experts are asked to make comparisons between pairwise criteria in the form of LNNs. Consequently, the linguistic neutrosophic preference relations can be established, as shown in **Table 4**. Suppose *x* = *y* = 1, based on Eq (41), the overall preference degrees of criteria are computed as: $r_{1}=(t_{3.1627},t_{1.6439},t_{2.0986})$, $r_{2}=(t_{4.6133},t_{2.0986},t_{1.4536})$, $r_{3}=(t_{1.7754},t_{3.5866},t_{2.6811})$, $r_{4}=(t_{2.4134},t_{2.6811},t_{3.7835})$ and $r_{5}=(t_{3.5317},t_{3.9330},t_{4.2246})$. Thereafter, using Eq (42), the weight of each evaluation criterion is: *w*_1_≈0.241, *w*_2_≈0.276, *w*_3_≈0.159, *w*_4_≈0.168 and *w*_5_≈0.156.

**Table 4:** Linguistic neutrosophic preference relations *R*.

R	*Q*_1_	*Q*_2_	*Q*_3_	*Q*_4_	*Q*_5_
*Q*_1_	-	(*t*_1_,*t*_1_,*t*_4_)	(*t*_4_,*t*_1_,*t*_2_)	(*t*_4_,*t*_2_,*t*_1_)	(*t*_3_,*t*_3_,*t*_2_)
*Q*_2_	(*t*_5_,*t*_5_,*t*_2_)	-	(*t*_5_,*t*_2_,*t*_1_)	(*t*_5_,*t*_1_,*t*_1_)	(*t*_3_,*t*_2_,*t*_2_)
*Q*_3_	(*t*_2_,*t*_5_,*t*_4_)	(*t*_1_,*t*_4_,*t*_5_)	-	(*t*_2_,*t*_4_,*t*_2_)	(*t*_2_,*t*_2_,*t*_1_)
*Q*_4_	(*t*_2_,*t*_4_,*t*_5_)	(*t*_1_,*t*_5_,*t*_5_)	(*t*_4_,*t*_2_,*t*_4_)	-	(*t*_2_,*t*_1_,*t*_2_)
*Q*_5_	(*t*_3_,*t*_3_,*t*_4_)	(*t*_3_,*t*_4_,*t*_4_)	(*t*_4_,*t*_4_,*t*_5_)	(*t*_4_,*t*_5_,*t*_4_)	-

In Phase III, the alternative rankings are determined. First, the comprehensive evaluation values are computed using the WLNEHM operator. Let *x* = *y* = 1, based on Eq (43), the comprehensive evaluation values of four sports attractions are computed as: $C_{1}=(t_{1.1369},t_{4.8869},t_{4.9654})$, $C_{2}=(t_{1.1433},t_{4.7470},t_{4.9739})$, $C_{3}=(t_{1.0270},t_{5.1035},t_{4.8574})$ and $C_{4}=(t_{0.8798},t_{4.9838},t_{4.9739})$. Thereafter, the score function and accuracy function values are calculated. Based on Eq (19), the score function values are calculated as: *S*(*C*_1_) = 0.1825, *S*(*C*_2_) = 0.1901, *S*(*C*_3_) = 0.1703 and *S*(*C*_4_) = 0.1623, and based on Eq (20), the accuracy function values are calculated as: *A*(*C*_1_) = −0.6381, *A*(*C*_2_) = −0.6384, *A*(*C*_3_) = −0.6384 and *A*(*C*_4_) = −0.6824. Last, the optimal alternative is attained. Because *S*(*C*_2_)>*S*(*C*_1_)>*S*(*C*_3_)>*S*(*C*_4_), the ranking order of four attractions is *P*_2_≻*P*_1_≻*P*_3_≻*P*_4_ and the most satisfied sports attraction is *P*_2_.

## Analyses and discussions

In this section, comparative and sensitivity analyses are conducted to testify the practicability and effectiveness of our method. Thereafter, some managerial insights and implications are provided.

### Comparative analyses

To clarify the practicality and efficiency of our method, comparative analyses are made from two perspectives. On the one hand, typical features of the proposed method and other existing approaches are described to show the suitability of our method. On the other hand, ranking results under dissimilar situations are analyzed to illustrate the advantages of the proposed approach.

#### (1) Characteristic comparison with existing methods

The different characteristic comparison results among different methods in existing literature and the proposed method are shown in **Table 5**.

**Table 5:** Characteristic comparison results with existing methods.

Literature	methods	Whether consider interrelationships of inputs	Whether consider interrelationships of inputs and itself	Type of operations
Fang and Ye [34]	LNWAA/LNWGA operators	No	No	Algebraic operations
Fan et al. [46]	LNNWBM/LNNWGBM operators	Yes	No	Algebraic operations
Liang et al. [47]	LNIGWHM/ LNIGGWHM operators	Yes	Yes	Algebraic operations
Liu and You [63]	Partitioned LNMSM operators	Yes	No	Algebraic
Luo et al. [48]	LNMSM operators	Yes	No	Algebraic operations
This study	LNEHM/WLNEHM operators	Yes	Yes	Einstein operations

From **Table 5**, it can be seen that compared with the methods based on LNWAA/LNWGA operators [34], our method can consider the interrelationships of inputs; compared with the methods in [46, 48, 63], the interactions of input arguments and itself are also taken into account in the proposed approach; and compared with the methods based on LNIGWHM/ LNIGGWHM operators [47], the Einstein operations of LNNs are adopted in our method to improve its flexibility in practice.

#### (2) Ranking comparison with existing methods

On the one hand, the same example in “Case study” Section is resolved with existing linguistic neutrosophic decision-making methods based on dissimilar aggregation operators. On the other hand, the proposed method is adopted to solve various linguistic neutrosophic decision-making problems in existing literature.

**Fig 2:**
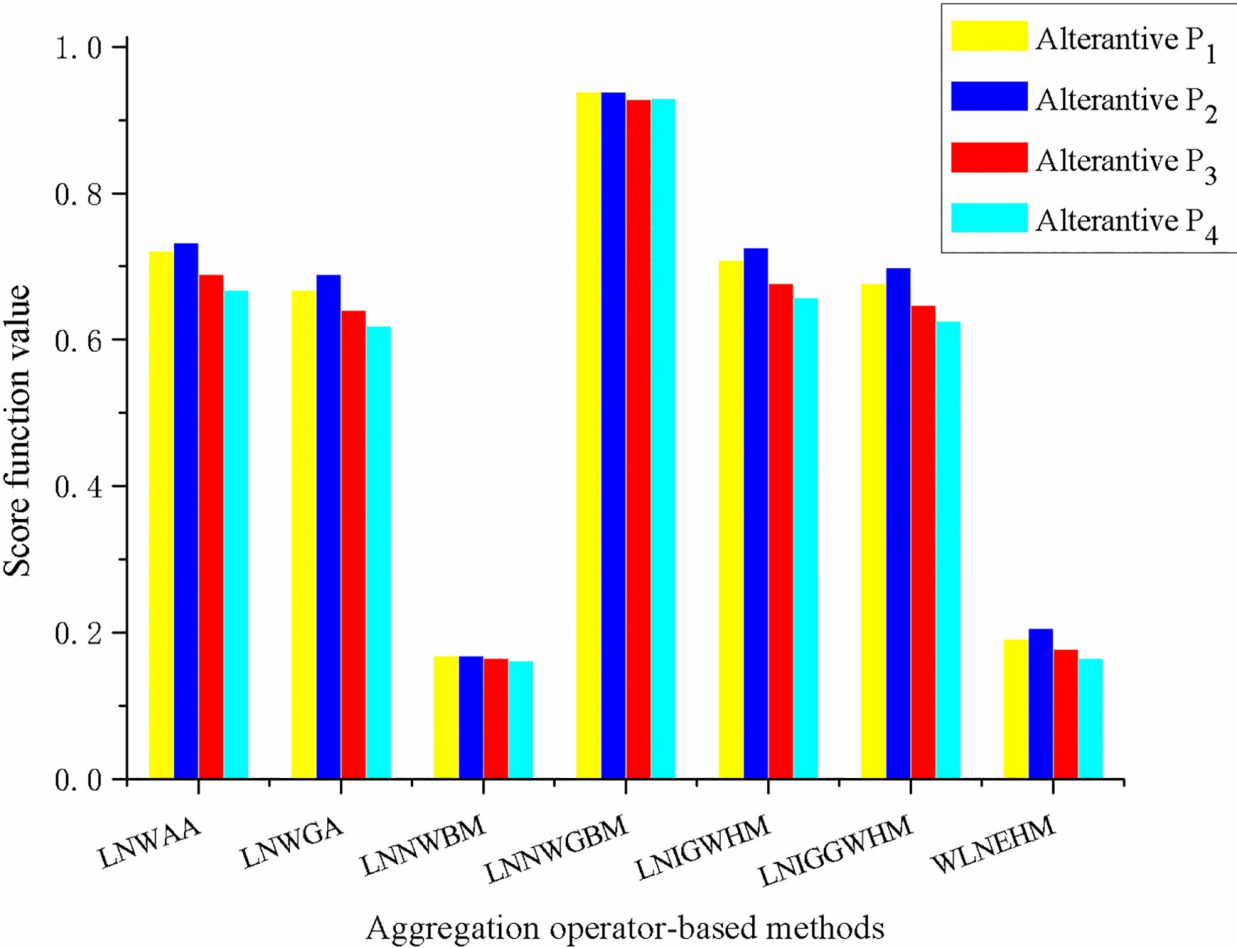
Score function values of four alternatives using different methods.

First, the score function values of four alternatives using different aggregation operator-based methods are depicted in **Fig 2**, and the detailed comparison results are described in **Table 6**.

**Table 6:** Ranking comparison results with aggregation operator-based methods under a same example.

Method	Score/Accuracy function value	Ranking order	The optimal alternative
Method with LNWAA operator [34]	*S*(*C*_1_) = 0.7206, *S*(*C*_2_) = 0.7325, *S*(*C*_3_) = 0.6891, *S*(*C*_4_) = 0.6670.	*P*_2_≻*P*_1_≻*P*_3_≻*P*_4_	*P*_2_
Method with LNWGA operator [34]	*S*(*C*_1_) = 0.6681, *S*(*C*_2_) = 0.6892, *S*(*C*_3_) = 0.6405, *S*(*C*_4_) = 0.6188.	*P*_2_≻*P*_1_≻*P*_3_≻*P*_4_	*P*_2_
Method with LNNWBM operator [46]	*S*(*C*_1_) = 0.1677, *S*(*C*_2_) = 0.1677, *S*(*C*_3_) = 0.1648, *S*(*C*_4_) = 0.1610; *A*(*C*_1_) = −0.6576, *A*(*C*_2_) = −0.6509.	*P*_2_≻*P*_1_≻*P*_3_≻*P*_4_	*P*_2_
Method with LNNWGBM operator [46]	*S*(*C*_1_) = 0.9383, *S*(*C*_2_) = 0.9383, *S*(*C*_3_) = 0.9282, *S*(*C*_4_) = 0.9291; *A*(*C*_1_) = 0.8874, *A*(*C*_2_) = 0.8772.	*P*_1_≻*P*_2_≻*P*_4_≻*P*_3_	*P*_1_
Method with LNIGWHM operator [47]	*S*(*C*_1_) = 0.7086, *S*(*C*_2_) = 0.7252, *S*(*C*_3_) = 0.6765, *S*(*C*_4_) = 0.6568.	*P*_2_≻*P*_1_≻*P*_3_≻*P*_4_	*P*_2_
Method with LNIGGWHM operator [47]	*S*(*C*_1_) = 0.6770, *S*(*C*_2_) = 0.6986, *S*(*C*_3_) = 0.6472, *S*(*C*_4_) = 0.6258.	*P*_2_≻*P*_1_≻*P*_3_≻*P*_4_	*P*_2_
The proposed method with WLNEHM operator	*S*(*C*_1_) = 0.1912, *S*(*C*_2_) = 0.2056, *S*(*C*_3_) = 0.1771, *S*(*C*_4_) = 0.1641.	*P*_2_≻*P*_1_≻*P*_3_≻*P*_4_	*P*_2_

From **Fig 2** and **Table 6**, it can be seen that more than 85 percent of approaches obtain the same optimal alternative *P*_2_, which is the same with that of the proposed method. Furthermore, the ranking orders with the proposed method and most of other existing methods based on dissimilar aggregation operators are the same (namely, *P*_2_≻*P*_1_≻*P*_3_≻*P*_4_). When coping with the illustrative example in Section 5 with the method based on the LNNWGBM operator [46], the score function values of alternatives *P*_1_ and *P*_2_ are equal (namely, *S*(*C*_1_) = *S*(*C*_2_)), the accuracy function values of *P*_1_ and *P*_2_ are near (*A*(*C*_1_) = 0.8874 and *A*(*C*_2_) = 0.8772), and the score function values of alternatives *P*_3_ and *P*_4_ are also near (*S*(*C*_3_) = 0.9282 and *S*(*C*_4_) = 0.9291). In this sense, the differences of the evaluation results between our method and the method based on the LNNWGBM operator [46] can be regarded as slight. Overall, our method, which is based on the Einstein operations and Heronian mean operators with two parameters, is more flexible and can simultaneously reflect the interactions of criteria and three membership degrees in LNNs.

On the other hand, the ranking orders under different examples are listed in **Table 7**.

**Table 7:** Ranking comparison results under different examples.

Decision-making problem	Ranking order with method in aboriginal literature	Ranking order with our method	Whether the same optimal alternative is obtained
Example in literature [34]	ϒ_4_≻ϒ_2_≻ϒ_3_≻ϒ_1_	ϒ_4_≻ϒ_2_≻ϒ_3_≻ϒ_1_	Yes
Example in literature [46]	*A*_4_≻*A*_2_≻*A*_3_≻*A*_1_	*A*_4_≻*A*_2_≻*A*_3_≻*A*_1_	Yes
Example in literature [47]	*x*_1_≻*x*_3_≻*x*_2_≻*x*_5_≻*x*_4_	*x*_1_≻*x*_4_≻*x*_2_≻*x*_3_≻*x*_5_	Yes
Example in literature [63]	*A*_1_≻*A*_2_≻*A*_3_≻*A*_4_/*A*_1_≻*A*_2_≻*A*_4_≻*A*_3_	*A*_1_≻*A*_2_≻*A*_3_≻*A*_4_	Yes
Example in literature [48]	*C*_1_≻*C*_2_≻*C*_3_≻*C*_4_	*C*_1_≻*C*_2_≻*C*_3_≻*C*_4_	Yes

From **Table 7**, it is clear that whether using the proposed method or the method in aboriginal literature, the same optimal alternative is always obtained in different examples. Furthermore, same evaluation results can be obtained in most cases when solving the decision-making problems with the proposed method and the corresponding aboriginal literature [34, 46, 48, 63]. Although there are some differences between the decision-making result with method in literature [47] and that with our method, the best alternative can be still distinguished. It demonstrates the feasibility of our method to some extent. For example, when dealing with example in literature [47], the evaluation result with method in original literature is *x*_1_≻*x*_3_≻*x*_2_≻*x*_5_≻*x*_4_, while the evaluation result with our method is *x*_1_≻*x*_4_≻*x*_2_≻*x*_3_≻*x*_5_. These two ranking orders are dissimilar, but the best alternative is always alternative *x*_1_. The reason of their differences may be the dissimilarity of the operational rules of LNNs.

### Sensitivity analyses

**Fig 3:**
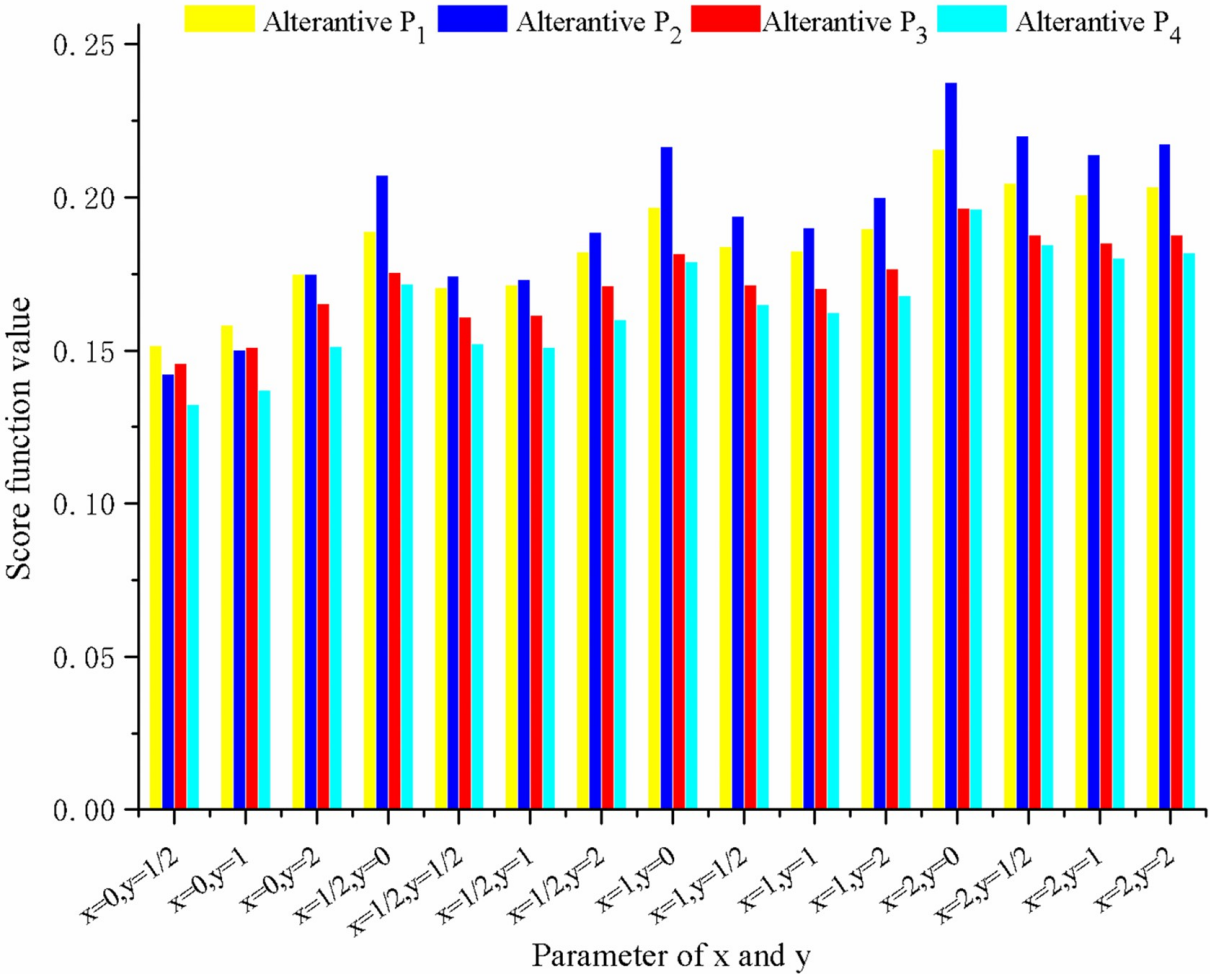
Score function values of four alternatives with different x and y values.

To check the influence of parameters *x* and *y* on the ranks of alternatives, different *x* and *y* values are assigned in Step 6 of the proposed method when solving the case in Section 6. Then, the score function values with different x and y values are depicted in **Fig 3**, and the ranking orders of alternatives are obtained under different situations, as shown in **Table 8**.

**Table 8:** Influence of *x* and *y* on the ranking orders.

*x* value	*y* value	Score function value	Ranking order
*x* = 0	*y* = 1/2	*S*(*C*_1_) = 0.1517,*S*(*C*_2_) = 0.1423,*S*(*C*_3_) = 0.1459,*S*(*C*_4_) = 0.1324.	*P*_1_≻*P*_3_≻*P*_2_≻*P*_4_
*x* = 0	*y* = 1	*S*(*C*_1_) = 0.1582,*S*(*C*_2_) = 0.1503,*S*(*C*_3_) = 0.1511,*S*(*C*_4_) = 0.1370.	*P*_1_≻*P*_3_≻*P*_2_≻*P*_4_
*x* = 0	*y* = 2	*S*(*C*_1_) = 0.1750,*S*(*C*_2_) = 0.1748,*S*(*C*_3_) = 0.1654,*S*(*C*_4_) = 0.1513.	*P*_1_≻*P*_2_≻*P*_3_≻*P*_4_
*x* = 1/2	*y* = 0	*S*(*C*_1_) = 0.1889,*S*(*C*_2_) = 0.2074,*S*(*C*_3_) = 0.1756,*S*(*C*_4_) = 0.1718.	*P*_2_≻*P*_1_≻*P*_3_≻*P*_4_
*x* = 1/2	*y* = 1/2	*S*(*C*_1_) = 0.1705,*S*(*C*_2_) = 0.1744,*S*(*C*_3_) = 0.1609,*S*(*C*_4_) = 0.1522.	*P*_2_≻*P*_1_≻*P*_3_≻*P*_4_
*x* = 1/2	*y* = 1	*S*(*C*_1_) = 0.1713,*S*(*C*_2_) = 0.1733,*S*(*C*_3_) = 0.1615,*S*(*C*_4_) = 0.1511.	*P*_2_≻*P*_1_≻*P*_3_≻*P*_4_
*x* = 1/2	*y* = 2	*S*(*C*_1_) = 0.1823,*S*(*C*_2_) = 0.1886,*S*(*C*_3_) = 0.1710,*S*(*C*_4_) = 0.1600.	*P*_2_≻*P*_1_≻*P*_3_≻*P*_4_
*x* = 1	*y* = 0	*S*(*C*_1_) = 0.1969,*S*(*C*_2_) = 0.2165,*S*(*C*_3_) = 0.1817,*S*(*C*_4_) = 0.1789.	*P*_2_≻*P*_1_≻*P*_3_≻*P*_4_
*x* = 1	*y* = 1/2	*S*(*C*_1_) = 0.1839,*S*(*C*_2_) = 0.1939,*S*(*C*_3_) = 0.1714,*S*(*C*_4_) = 0.1651.	*P*_2_≻*P*_1_≻*P*_3_≻*P*_4_
*x* = 1	*y* = 1	*S*(*C*_1_) = 0.1825,*S*(*C*_2_) = 0.1901,*S*(*C*_3_) = 0.1703,*S*(*C*_4_) = 0.1623.	*P*_2_≻*P*_1_≻*P*_3_≻*P*_4_
*x* = 1	*y* = 2	*S*(*C*_1_) = 0.1897,*S*(*C*_2_) = 0.1999,*S*(*C*_3_) = 0.1767,*S*(*C*_4_) = 0.1679.	*P*_2_≻*P*_1_≻*P*_3_≻*P*_4_
*x* = 2	*y* = 0	*S*(*C*_1_) = 0.2157,*S*(*C*_2_) = 0.2376,*S*(*C*_3_) = 0.1965,*S*(*C*_4_) = 0.1961.	*P*_2_≻*P*_1_≻*P*_3_≻*P*_4_
*x* = 2	*y* = 1/2	*S*(*C*_1_) = 0.2045,*S*(*C*_2_) = 0.2201,*S*(*C*_3_) = 0.1878,*S*(*C*_4_) = 0.1846.	*P*_2_≻*P*_1_≻*P*_3_≻*P*_4_
*x* = 2	*y* = 1	*S*(*C*_1_) = 0.2008,*S*(*C*_2_) = 0.2141,*S*(*C*_3_) = 0.1850,*S*(*C*_4_) = 0.1802.	*P*_2_≻*P*_1_≻*P*_3_≻*P*_4_
*x* = 2	*y* = 2	*S*(*C*_1_) = 0.2035,*S*(*C*_2_) = 0.2175,*S*(*C*_3_) = 0.1876,*S*(*C*_4_) = 0.1818.	*P*_2_≻*P*_1_≻*P*_3_≻*P*_4_

From **Fig 3** and **Table 8**, it can be determined that the score function values and ranking orders are dissimilar under different *x* and *y* values. With the growth of *y* value, the alteration of score function value is also inconsistent. However, the larger the value of *x*, the larger the score function value of each alternative. In most cases, the ranking order *P*_2_≻*P*_1_≻*P*_3_≻*P*_4_ is the same, which verifies the feasibility of our method. Thus, to simplify the calculation, simple *x* and *y* values can be chosen, such as *x* = *y* = 1. On the other hand, the interactions of alternatives are highlighted with the increase of *x* and *y* values. Thus, various combinations of *x* and *y* values can be selected to make the decision-making process more flexible.

In summary, the main merits of the proposed method are: First, the relationships among different evaluation dimensions can be captured based on the Heronian mean operators. Second, the interactions of inputs and itself are also considered without redundant calculations. Consequently, the interrelations of input arguments and multiple membership degrees in LNNs can be simultaneously explored. Third, the defined linguistic neutrosophic Einstein operations with two parameters improve the flexibility and smooth of our method.

### Managerial insights and implications

The proposed methods based on LNEHM and WLNEHM mean operators offer meaningful instructions for the sustainability evaluation of sports tourism. According to the comparative and sensitivity analyses, several managerial insights and implications are acquired as follows:

1. In the practice of sustainable development of sports tourism, all evaluation criteria should be considered comprehensively. Economy and sociality are identified as the most important criteria during the sustainability evaluation of sports tourism. However, the importance of other dimensions, such as institution, environment and technology, cannot be ignored as well. Environmental and institutional aspects should be integrated into sustainable development decision-making and corresponding technology need to be developed.
2. There are great interconnection and interdependence among five different dimensions of sustainability evaluation in sports tourism. For example, the realization of environmental goals needs some technical and institutional support. Comparative and sensitivity analyses indicate that the proposed method can reflect the interrelationships among inputs without redundant calculations and possesses superior flexibility and smoothness, so as to assist decision-makers to be more systematic and facilitate the evaluation process.
3. Sports tourism has the basic attribute of sustainable development. Through the establishment of a cooperative governance system of sports tourism, local residents can be promoted to participate in sports tourism services. Policymakers can adopt environmental protection measures and information and communication technology to achieve sustainable development of sports tourism in policy, social culture, tourism economy, environment, technology and other levels.

## Conclusions

Sustainable sports tourism is of great significance for promoting the quality and efficiency of sports industry, fostering new momentum and expanding new space for economic development. This study focused on the sustainability evaluation of sports tourism with a novel fuzzy decision-making support framework. For this purpose, LNNs were adopted to describe complicated fuzzy evaluation data and an entire evaluation index system was established with newfound assessment criteria. As a result, their interactions were identified with new proposed aggregation operators, called “LNEHM operator” and “WLNEHM operator”. At the same time, some key properties of the LNEHM and WLNEHM operators were justified and the influences of two parameters were analyzed. Moreover, a new MCDM framework was proposed to address multi-criteria evaluation issues in linguistic neutrosophic circumstances. A case with in-depth discussions was investigated to demonstrate the practicability and availability of our method. In the end, the case study and analyses indicated that the proposed approach was workable and beneficial for the sustainability evaluation of sports tourism, and could offer guidance for the management and selection of sports attractions.

However, this study is still subject to several limitations. For example, the criteria weight is subjective with preference relations technology, the importance degrees of experts were assumed equal, and the limited rationality of decision makers were ignored. In the future, objective criteria weights should be added and experts’ weight calculation models can be considered. Second, the consistency and the consensus problems in group decision-making environments are worthy to be studied [64–66]. Furthermore, the proposed approach can be improved by considering the limited rationality or psychological characteristics of decision makers during the evaluation process.

## Supporting information

S1 Appendix Table A1. Linguistic neutrosophic decision-making methods and their applications.
(DOCX)

S2 Appendix Proof of Theorem 2. (DOCX)
