# Supplementary material for: Sustainability evaluation of sports tourism using a linguistic neutrosophic multi-criteria decision-making method
Source: PLoS One. 2024 Mar 18;19(3):e0300341. doi: 10.1371/journal.pone.0300341 (PMC10947702; doi:10.1371/journal.pone.0300341)
Supplement: S1 Appendix — Linguistic neutrosophic decision-making methods and their applications. (DOCX) [file pone.0300341.s001.docx]

**Appendix A**

**Table A1** Linguistic neutrosophic decision-making methods and their applications

| Literature | Methods | Criteria weight calculation | Interactions among inputs | Applications |
| --- | --- | --- | --- | --- |
| Li et al. [1 | EDAS | Yes | No | Property management companies selection problem |
| Garg [2] | COPRAS | Yes | No | Information technology outsourcing selection |
| Liu and You [3] | Bidirectional projection-based | Yes | Yes | Investment risk evaluation, investment alternatives selection, career development |
| Wang et al. [4] | VIKOR | Yes | No | Fault handling point selection problem |
| Zhu et al. [5] | Regret theory, PROMETHEE | Yes | No | Failure modes risk priority assessment |
| Bhaumik et al. [6] | Multi-objective matrix game | No | No | Tourism management |
| Zhang et al. [7] | Partial Hausdorff distance | No | No | Auto part selection problem |
| Fang and Ye [8] | LNWAA, LNWGA | No | No | Investment alternatives selecting problem |
| Liu and You [9] | Hamy mean operators | Yes | Yes | Investment alternatives selecting problem |
| Fan et al. [10] | LNNWBM, LNNWGBM | No | Yes | Investment alternatives selecting problem |
| Liang et al. [11] | LNIWHM, LNIGWHM | Yes | No | The optimal mining method selection problem |
| Luo et al. [12] | Maclaurin symmetric mean operators | No | Yes | Performance evaluation of human resources |
| Li et al. [13] | Weight Muirhead Mean operators | No | Yes | Allocate reliability indicators in engineering fields |
| Chen et al. [14] | Generalized weighted Choquet Heronian mean operator | No | Yes | Low-carbon suppliers selection problem |
| Fan et al. [15] | Weighted Heronian mean operators | No | Yes | Car purchase decision-making problem |
| Liu et al. [16] | Power Heronian aggregation operators | No | Yes | Air quality evaluation |
| Wang and Liu [17] | Generalized partitioned Bonferroni mean operators | No | Yes | Green suppliers selection problem |
| Li et al. [18] | Geometric Heronian mean operators | No | Yes | Low-carbon supplier selection problem |
| Pamučar et al. [19] | COmbinative Distance-based ASsessment (CODAS) method | Yes | No | Power-generation technology selection |
| Shi and Ye [20] | Cosine measures-based method | No | No | Investment alternatives selecting problem |

**References**

[1] Li, Y. Y., Wang, J. Q., & Wang, T. L. (2019). A linguistic neutrosophic multi-criteria group decision-making approach with EDAS method. Arabian Journal for Science and Engineering, 44(3), 2737-2749.

[2] Garg, H. (2019). Algorithms for possibility linguistic single-valued neutrosophic decision-making based on COPRAS and aggregation operators with new information measures. Measurement, 138, 278-290.

[3] Liu, P., & You, X. (2019). Bidirectional projection measure of linguistic neutrosophic numbers and their application to multi-criteria group decision making. Computers & Industrial Engineering, 128, 447-457.

[4] Wang, X., Geng, Y., Yao, P., & Yang, M. (2019). Multiple attribute group decision making approach based on extended VIKOR and linguistic neutrosophic set. Journal of Intelligent & Fuzzy Systems, 36(1), 149-160.

[5] Zhu, J., Shuai, B., Li, G., Chin, K. S., & Wang, R. (2020). Failure mode and effect analysis using regret theory and PROMETHEE under linguistic neutrosophic context. Journal of Loss Prevention in the Process Industries, 64, 104048.

[6] Bhaumik, A., Roy, S. K., & Weber, G. W. (2021). Multi-objective linguistic-neutrosophic matrix game and its applications to tourism management. Journal of Dynamics & Games, 8(2), 101-118.

[7] Zhang, L., Zhang, C., Tian, G., Chen, Z., Fathollahi-Fard, A. M., Zhao, X., & Wong, K. Y. (2023). A multi-criteria group-based decision-making method considering linguistic neutrosophic clouds. Expert Systems with Applications, 226, 119936.

[8] Fang, Z., & Ye, J. (2017). Multiple attribute group decision-making method based on linguistic neutrosophic numbers. Symmetry, 9(7), 111.

[9] Liu, P., & You, X. (2018). Some linguistic neutrosophic Hamy mean operators and their application to multi-attribute group decision making. PloS one, 13(3), e0193027.

[10] Fan, C., Ye, J., Hu, K., & Fan, E. (2017). Bonferroni mean operators of linguistic neutrosophic numbers and their multiple attribute group decision-making methods. Information, 8(3), 107.

[11] Liang, W., Zhao, G., & Hong, C. (2019). Selecting the optimal mining method with extended multi-objective optimization by ratio analysis plus the full multiplicative form (MULTIMOORA) approach. Neural Computing and Applications, 31(10), 5871-5886.

[12] Luo, S. Z., Xing, L. N., & Ren, T. (2022). Performance evaluation of human resources based on linguistic neutrosophic Maclaurin Symmetric mean operators. Cognitive Computation, 14(2), 547-562.

[13] Li, G., Zhong, Y., Chen, C., Jin, T., & Liu, Y. (2022). Reliability allocation method based on linguistic neutrosophic numbers weight Muirhead mean operator. Expert Systems with Applications, 193, 116504.

[14] Chen, T., He, S. S., Wang, J. Q., Li, L., & Luo, H. (2019). Novel operations for linguistic neutrosophic sets on the basis of Archimedean copulas and co-copulas and their application in multi-criteria decision-making problems. Journal of Intelligent & Fuzzy Systems, 37(2), 2887-2912.

[15] Fan, C., Hu, K., Feng, S., Ye, J., & Fan, E. (2019). Heronian mean operators of linguistic neutrosophic multisets and their multiple attribute decision-making methods. International Journal of Distributed Sensor Networks, 15(4), 1550147719843059.

[16] Liu, P., Mahmood, T., & Khan, Q. (2018). Group decision making based on power Heronian aggregation operators under linguistic neutrosophic environment. International Journal of Fuzzy Systems, 20, 970-985.

[17] Wang, Y., & Liu, P. (2018). Linguistic neutrosophic generalized partitioned Bonferroni mean operators and their application to multi-attribute group decision making. Symmetry, 10(5), 160.

[18] Li, Y. Y., Zhang, H., & Wang, J. Q. (2017). Linguistic neutrosophic sets and their application in multicriteria decision-making problems. International Journal for Uncertainty Quantification, 7(2).

[19] Pamučar, D., Badi, I., Sanja, K., & Obradović, R. (2018). A novel approach for the selection of power-generation technology using a linguistic neutrosophic CODAS method: A case study in Libya. Energies, 11(9), 2489.

[20] Shi, L., & Ye, J. (2017). Cosine measures of linguistic neutrosophic numbers and their application in multiple attribute group decision-making. Information, 8(4), 117.
