# Supplementary material for: Sustainability evaluation of sports tourism using a linguistic neutrosophic multi-criteria decision-making method
Source: PLoS One. 2024 Mar 18;19(3):e0300341. doi: 10.1371/journal.pone.0300341 (PMC10947702; doi:10.1371/journal.pone.0300341)
Supplement: S2 Appendix — (DOCX) [file pone.0300341.s002.docx]

**Appendix B**

**Proof of Theorem 2.**

Based on Definition 6, we have $LNEHM^{x,y}(\eta_{1},\eta_{2},\cdots,\eta_{u})=\left(\frac{2}{u(u+1)}\sum_{i=1,j=i}^{u} (\eta_{i}^{x}\otimes_{E}\eta_{j}^{y})\right)^{\frac{1}{x+y}}$.

On right-hand sides, according to Eq. (8), we have $\eta_{i}^{x}=(t_{a_{i}},t_{b_{i}},t_{c_{i}})^{x}=(t_{\frac{2v\alpha_{i}^{x}}{(2-\alpha_{i})^{x}+\alpha_{i}^{x}}},t_{v\cdot\frac{(1+\beta_{i})^{x}-(1-\beta_{i})^{x}}{(1+\beta_{i})^{x}+(1-\beta_{i})^{x}}},t_{v\cdot\frac{(1+\gamma_{i})^{x}-(1-\gamma_{i})^{x}}{(1+\gamma_{i})^{x}+(1-\gamma_{i})^{x}}})$ and $\eta_{j}^{y}=(t_{a_{j}},t_{b_{j}},t_{c_{j}})^{y}=(t_{\frac{2v\alpha_{j}^{y}}{(2-\alpha_{j})^{y}+\alpha_{j}^{y}}},t_{v\cdot\frac{(1+\beta_{j})^{y}-(1-\beta_{j})^{y}}{(1+\beta_{j})^{y}+(1-\beta_{j})^{y}}},t_{v\cdot\frac{(1+\gamma_{j})^{y}-(1-\gamma_{j})^{y}}{(1+\gamma_{j})^{y}+(1-\gamma_{j})^{y}}})$.

Then, based on Eq. (6), we have $\begin{matrix} \eta_{i}^{x}\otimes_{E}\eta_{j}^{y}=(t_{\frac{v\cdot\frac{2v\alpha_{i}^{x}}{(2-\alpha_{i})^{x}+\alpha_{i}^{x}}\cdot\frac{2v\alpha_{j}^{y}}{(2-\alpha_{j})^{y}+\alpha_{j}^{y}}}{1+(1-\frac{2v\alpha_{i}^{x}}{(2-\alpha_{i})^{x}+\alpha_{i}^{x}})(1-\frac{2v\alpha_{j}^{y}}{(2-\alpha_{j})^{y}+\alpha_{j}^{y}})}},t_{\frac{v(v\cdot\frac{(1+\beta_{i})^{x}-(1-\beta_{i})^{x}}{(1+\beta_{i})^{x}+(1-\beta_{i})^{x}}+v\cdot\frac{(1+\beta_{j})^{y}-(1-\beta_{j})^{y}}{(1+\beta_{j})^{y}+(1-\beta_{j})^{y}})}{1+v\cdot\frac{(1+\beta_{i})^{x}-(1-\beta_{i})^{x}}{(1+\beta_{i})^{x}+(1-\beta_{i})^{x}}\cdot v\cdot\frac{(1+\beta_{j})^{y}-(1-\beta_{j})^{y}}{(1+\beta_{j})^{y}+(1-\beta_{j})^{y}}}},t_{\frac{v(v\cdot\frac{(1+\gamma_{i})^{x}-(1-\gamma_{i})^{x}}{(1+\gamma_{i})^{x}+(1-\gamma_{i})^{x}}+v\cdot\frac{(1+\gamma_{j})^{y}-(1-\gamma_{j})^{y}}{(1+\gamma_{j})^{y}+(1-\gamma_{j})^{y}})}{1+v\cdot\frac{(1+\gamma_{i})^{x}-(1-\gamma_{i})^{x}}{(1+\gamma_{i})^{x}+(1-\gamma_{i})^{x}}\cdot v\cdot\frac{(1+\gamma_{j})^{y}-(1-\gamma_{j})^{y}}{(1+\gamma_{j})^{y}+(1-\gamma_{j})^{y}}}}) \\ \text{ }=(t_{\frac{2v\alpha_{i}^{x}\alpha_{j}^{y}}{(2-\alpha_{i})^{x}(2-\alpha_{j})^{y}+\alpha_{i}^{x}\alpha_{j}^{y}}},t_{v\cdot\frac{(1+\beta_{i})^{x}(1+\beta_{j})^{y}-(1-\beta_{i})^{x}(1-\beta_{j})^{y}}{(1+\beta_{i})^{x}(1+\beta_{j})^{y}+(1-\beta_{i})^{x}(1-\beta_{j})^{y}}},t_{v\cdot\frac{(1+\gamma_{i})^{x}(1+\gamma_{j})^{y}-(1-\gamma_{i})^{x}(1-\gamma_{j})^{y}}{(1+\gamma_{i})^{x}(1+\gamma_{j})^{y}+(1-\gamma_{i})^{x}(1-\gamma_{j})^{y}}}) \end{matrix}$.

Thus, according to Eq. (1) and Eq. (7), $\frac{2}{u(u+1)}\sum_{i=1,j=i}^{u} (\eta_{i}^{x}\otimes_{E}\eta_{j}^{y})=$ $\left(t_{v\cdot\frac{1-\left(\prod_{i=1,j=i}^{u} \frac{(2-\alpha_{i})^{x}(2-\alpha_{j})^{y}-\alpha_{i}^{x}\alpha_{j}^{y}}{(2-\alpha_{i})^{x}(2-\alpha_{j})^{y}+3\alpha_{i}^{x}\alpha_{j}^{y}}\right)^{\frac{2}{u(u+1)}}}{1+\left(\prod_{i=1,j=i}^{u} \frac{(2-\alpha_{i})^{x}(2-\alpha_{j})^{y}-\alpha_{i}^{x}\alpha_{j}^{y}}{(2-\alpha_{i})^{x}(2-\alpha_{j})^{y}+3\alpha_{i}^{x}\alpha_{j}^{y}}\right)^{\frac{2}{u(u+1)}}}},t_{\frac{2v}{1+\left(\prod_{i=1,j=i}^{u} \frac{(1+\beta_{i})^{x}(1+\beta_{j})^{y}+3(1-\beta_{i})^{x}(1-\beta_{j})^{y}}{(1+\beta_{i})^{x}(1+\beta_{j})^{y}-(1-\beta_{i})^{x}(1-\beta_{j})^{y}}\right)^{\frac{2}{u(u+1)}}}},t_{\frac{2v}{1+\left(\prod_{i=1,j=i}^{u} \frac{(1+\gamma_{i})^{x}(1+\gamma_{j})^{y}+3(1-\gamma_{i})^{x}(1-\gamma_{j})^{y}}{(1+\gamma_{i})^{x}(1+\gamma_{j})^{y}-(1-\gamma_{i})^{x}(1-\gamma_{j})^{y}}\right)^{\frac{2}{u(u+1)}}}}\right)$.

Let $\alpha=\left(\prod_{i=1,j=i}^{u} \frac{(2-\alpha_{i})^{x}(2-\alpha_{j})^{y}-\alpha_{i}^{x}\alpha_{j}^{y}}{(2-\alpha_{i})^{x}(2-\alpha_{j})^{y}+3\alpha_{i}^{x}\alpha_{j}^{y}}\right)^{\frac{2}{u(u+1)}}$, $\beta=\left(\prod_{i=1,j=i}^{u} \frac{(1+\beta_{i})^{x}(1+\beta_{j})^{y}+3(1-\beta_{i})^{x}(1-\beta_{j})^{y}}{(1+\beta_{i})^{x}(1+\beta_{j})^{y}-(1-\beta_{i})^{x}(1-\beta_{j})^{y}}\right)^{\frac{2}{u(u+1)}}$ and $\gamma=\left(\prod_{i=1,j=i}^{u} \frac{(1+\gamma_{i})^{x}(1+\gamma_{j})^{y}+3(1-\gamma_{i})^{x}(1-\gamma_{j})^{y}}{(1+\gamma_{i})^{x}(1+\gamma_{j})^{y}-(1-\gamma_{i})^{x}(1-\gamma_{j})^{y}}\right)^{\frac{2}{u(u+1)}}$, then $\frac{2}{u(u+1)}\sum_{i=1,j=i}^{u} (\eta_{i}^{x}\otimes_{E}\eta_{j}^{y})=(t_{v\cdot\frac{1-\alpha}{1+\alpha}},t_{\frac{2v}{1+\beta}},t_{\frac{2v}{1+\gamma}})$.

Therefore, based on Eq. (8), $\begin{matrix} \text{ }\left(\frac{2}{u(u+1)}\sum_{i=1,j=i}^{u} (\eta_{i}^{x}\otimes_{E}\eta_{j}^{y})\right)^{\frac{1}{x+y}}=(t_{v\cdot\frac{1-\alpha}{1+\alpha}},t_{\frac{2v}{1+\beta}},t_{\frac{2v}{1+\gamma}})^{\frac{1}{x+y}} \\ =(t_{\frac{2v\cdot(v\cdot\frac{1-\alpha}{1+\alpha})^{\frac{1}{x+y}}}{(2-v\cdot\frac{1-\alpha}{1+\alpha})^{\frac{1}{x+y}}+(v\cdot\frac{1-\alpha}{1+\alpha})^{\frac{1}{x+y}}}},t_{v\cdot\frac{(1+\frac{2v}{1+\beta})^{\frac{1}{x+y}}-(1-\frac{2v}{1+\beta})^{\frac{1}{x+y}}}{(1+\frac{2v}{1+\beta})^{\frac{1}{x+y}}+(1-\frac{2v}{1+\beta})^{\frac{1}{x+y}}}},t_{v\cdot\frac{(1+\frac{2v}{1+\gamma})^{\frac{1}{x+y}}-(1-\frac{2v}{1+\gamma})^{\frac{1}{x+y}}}{(1+\frac{2v}{1+\gamma})^{\frac{1}{x+y}}+(1-\frac{2v}{1+\gamma})^{\frac{1}{x+y}}}})=\left(t\frac{2v}{1+\left(\frac{1+3\alpha}{1-\alpha}\right)^{\frac{1}{x+y}}},t_{v\cdot\frac{1-\left(\frac{\beta-1}{\beta+3}\right)^{\frac{1}{x+y}}}{1+\left(\frac{\beta-1}{\beta+3}\right)^{\frac{1}{x+y}}}},t_{v\cdot\frac{1-\left(\frac{\gamma-1}{\gamma+3}\right)^{\frac{1}{x+y}}}{1+\left(\frac{\gamma-1}{\gamma+3}\right)^{\frac{1}{x+y}}}}\right) \end{matrix}$.

Hence, we have $LNEHM^{x,y}(\eta_{1},\eta_{2},\cdots,\eta_{u})=\left(\frac{2}{u(u+1)}\sum_{i=1,j=i}^{u} (\eta_{i}^{x}\otimes_{E}\eta_{j}^{y})\right)^{\frac{1}{x+y}}=\left(t_{\frac{2v}{1+\left(\frac{1+3\alpha}{1-\alpha}\right)^{\frac{1}{x+y}}}},t_{v\cdot\frac{1-\left(\frac{\beta-1}{\beta+3}\right)^{\frac{1}{x+y}}}{1+\left(\frac{\beta-1}{\beta+3}\right)^{\frac{1}{x+y}}}},t_{v\cdot\frac{1-\left(\frac{\gamma-1}{\gamma+3}\right)^{\frac{1}{x+y}}}{1+\left(\frac{\gamma-1}{\gamma+3}\right)^{\frac{1}{x+y}}}}\right)$.

Now, the proof is completed.
